# Advances in the Fabrication of Nanoporous Anodic Aluminum Oxide and Its Applications to Sensors: A Review

**DOI:** 10.3390/nano13212853

**Published:** 2023-10-27

**Authors:** Chin-An Ku, Chung-Yu Yu, Chia-Wei Hung, Chen-Kuei Chung

**Affiliations:** Department of Mechanical Engineering, National Cheng Kung University, Tainan 701, Taiwan

**Keywords:** anodic aluminum oxide, AAO, nanofabrication, nanoporous, humidity, SERS, sensor

## Abstract

Nanoporous anodic aluminum oxide (AAO) is an important template for 1D nanomaterial synthesis. It is used as an etching template for nanopattern transfer in a variety of contexts, including nanostructured material synthesis, electrical sensors, optical sensors, photonic and electronic devices, photocatalysis, and hardness and anticorrosion improvement. In this review, we focus on various fabrication methods, pore geometry modification, and recent advances of AAO, as well as sensor applications linked to our environment, daily life, and safety. Pore geometry is concerned with the material composition, applied voltage mold, electrolyte type, temperature, and anodizing time during the fabrication of AAOs and for adjusting their pore size and profile. The applied voltage can be divided into four types: direct current anodization (DCA), reverse pulse anodization, pulse anodization (PA), and hybrid pulse anodization (HPA). Conventional AAOs are fabricated using DCA and mild anodization (MA) at a relatively low temperature (−5~15 °C) to reduce the Joule heating effect. Moreover, the issues of costly high-purity aluminum and a long processing time can be improved using HPA to diminish the Joule heating effect at relatively high temperatures of 20–30 °C with cheap low-purity (≤99%) aluminum. The AAO-based sensors discussed here are primarily divided into electrical sensors and optical sensors; the performance of both sensors is affected by the sensing material and pore geometry. The electrical sensor is usually used for humidity or gas measurement applications and has a thin metal film on the surface as an electrode. On the contrary, the AAO optical sensor is a well-known sensor for detecting various substances with four kinds of mechanisms: interference, photoluminescence, surface plasma resonance, and surface-enhanced Raman scattering (SERS). Especially for SERS mechanisms, AAO can be used either as a solid support for coating metal nanoparticles or a template for depositing the metal content through the nanopores to form the nanodots or nanowires for detecting substances. High-performance sensors will play a crucial role in our living environments and promote our quality of life in the future.

## 1. Overview of Nanoporous AAO and its Applications

Over the past decades, the fabrication of nanostructured materials has been a crucial topic of research because of their significant physical and chemical properties for global applications. For example, self-cleaning phenomena are due to the super-hydrophobic property of the nanostructured surface of the leaf, which is called the “lotus effect”. Materials with a microstructure of the characteristic length scale of typically 1–100 nm can be defined as nanostructured materials. This microstructure refers to the arrangement of the atoms or molecules, and the size of a phase in one, two, or three dimensions, to produce mesoscopic phenomena. Therefore, nanomaterials with controllable structures have attracted increasing attention in past decades due to their size effect, quantum tunneling effect, quantum confinement effect, and their specific surface area to produce distinctive and unique physical, mechanical, electrical, and chemical properties compared with traditional bulk and thick-film materials [1]. In general, the most common method for the top-down fabrication of nanostructured material with various elements is the photolithography patterning technique, followed by etching [2]. However, the requirement of costly equipment and strict environmental factors has become a problem, especially in the scale of several tens of nanometers. The laser ablation method [3] and the chemical vapor deposition (CVD) method [4] provide limited solutions for the fabrication of nanorods or nanotubes in specific materials, like carbon. However, geometric factors, like the diameter or aspect ratio, are difficult to control using these two technologies. Therefore, template-assisted deposition, growth, and synthesis using nanoporous anodic aluminum oxide (AAO) has been considered one of the most promising methods for fabricating 1D nanostructures.

**Figure 1:**
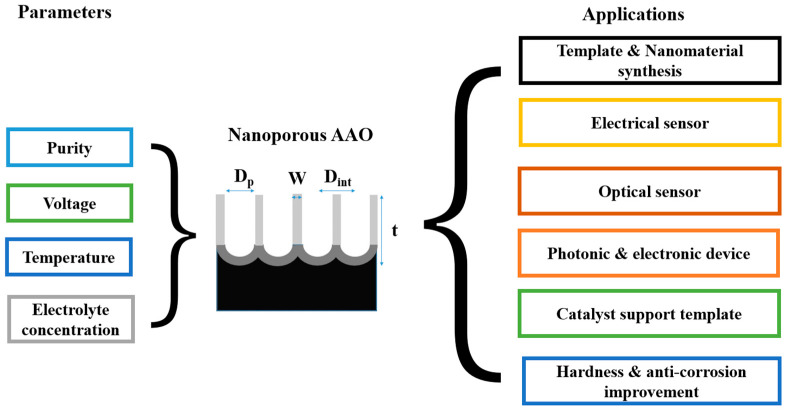
The anodization parameters and pore geometry overview of AAO and its applications. The interactive effects of parameters, profiles, and applications influence one another.

**Figure 2:**
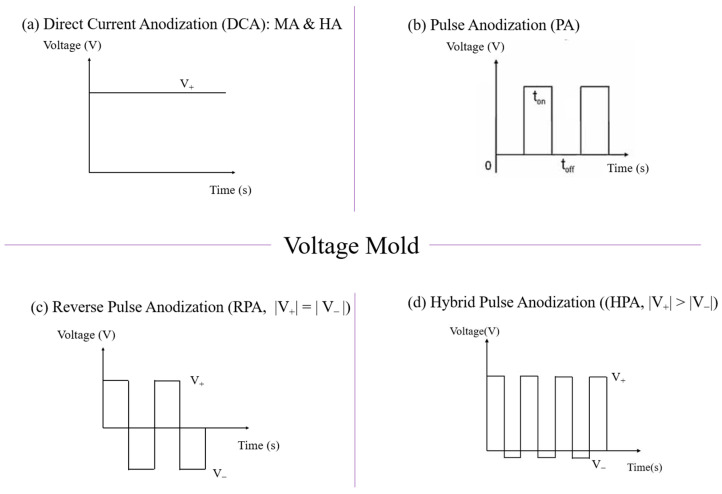
The voltage mold of AAO fabrication processes by different methods: (**a**) direct current anodization (DCA), (**b**) pulse anodization (PA) by a positive voltage and followed by 0 voltage, (**c**) reverse pulse anodization by a positive voltage and followed by the same value of negative voltage, and (**d**) hybrid pulse anodization (HPA) by a positive voltage and followed by a relatively small negative voltage.

**Figure 3:**
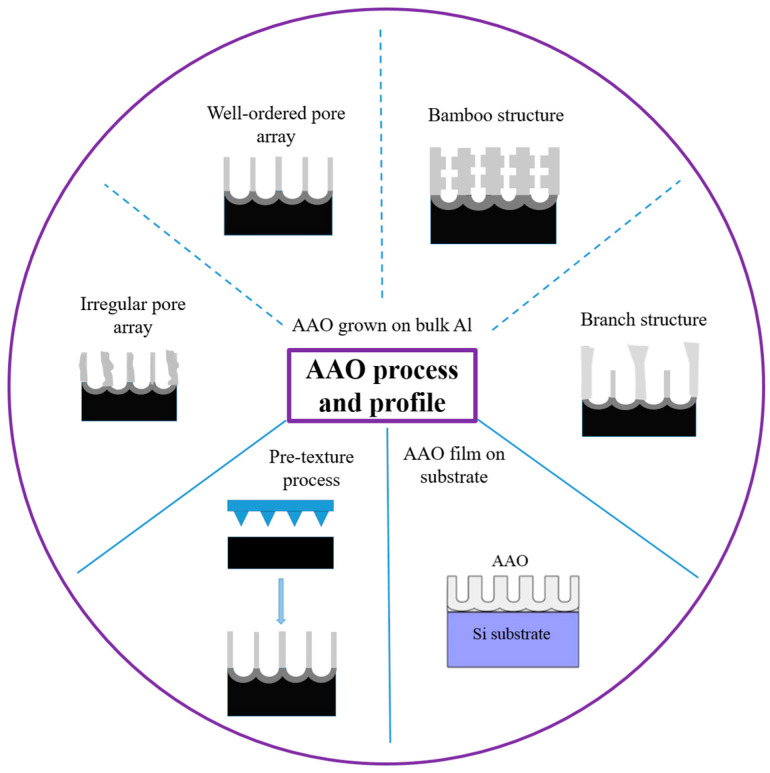
AAO process and profile control. AAO fabrication process can be divided into three categories: AAO grown on bulk Al, pretexture process, and AAO film on substrate.

AAO technology is one of the most well-known technologies for the fabrication of nanostructured templates in industry and scientific research. Compared with other nanomaterial templates, AAO exhibits the advantages of nanoscale pores, self-organization, tunable nanostructures, and good physical and chemical stability [5]. Further, aluminum is one of the most famously used metals in industry, which makes aluminum surface treatments highly compatible with production lines. These characteristics make AAO an object for extensive research. AAO is categorized into a barrier type and porous type, according to its structure. The barrier type of AAO, with a thin and compact structure, was largely used in the past for protection and dielectric capacitors [6]. By contrast, the porous type of AAO, with a highly ordered nanopore arrangement, has received increasing attention as a template for fabricating nanowires or synthesizing other 1D/2D nanostructures [6,7,8,9,10], and as a nanopattern transfer for micro/nano applications, for instance, in the triboelectric nanogenerator (TENG) field [11,12]. AAO, as a nanomaterial template, is fabricated using certain electrochemical processes. Figure 1 provides an overview of the anodization parameters with the dependent pore geometry and morphology of AAO, including the pore diameter (D_p_), interpore distance (D_int_), pore wall thickness (w), barrier thickness (t_b_), and AAO thickness (t). The fabrication parameters during this process are concerned with the formation of AAO’s geometry and profile, which are linked to various applications. AAO’s pore geometry and morphology are mainly affected by the purity, voltage and voltage mold, reaction temperature, electrolyte content and concentration, and time. Impurities in the Al substrate will increase the dissolution, which causes an irregular pore structure with a non-obvious hexagonal structure. The anodization voltage is proportional to the D_int_, with a positive relation to the D_p_. The proper anodization voltage required for AAO formation is dependent on the electrolyte type and concentration. Modulations in AAO’s structure occur through complex interactions, which will directly affect the application performance. The voltage mold of the AAO fabrication process is shown in Figure 2. Generally speaking, the production of AAO includes polishing and different parameter-controlled anodization processes to meet the required nanostructure. In the detailed electrochemical anodization at a certain electrolyte content, the parameters can be modulated, including the temperature (low temperature vs. high temperature), voltage (mild anodization (MA) vs. hard anodization (HA)), waveforms of direct current anodization (DCA, Figure 2a), pulse anodization (PA, Figure 2b), reverse pulse anodization (Figure 2c), and hybrid pulse anodization (HPA, Figure 2d), for the desired pore structure and a more efficient process [13,14,15,16,17,18,19,20,21,22]. Figure 3 shows the various schematic AAO profiles that can be modulated by different processes or voltage molds. The AAO fabrication process can be divided into three categories [23,24]. The first is the most well-known process of fabricating AAO on bulk Al; this is presently the most useful method for preparing AAO. The special structure of bamboo [25,26] and branch structure [27,28] are also fabricated from bulk Al using the MA and HA modulation methods. The second is the pretextured stamp nanoimprinting method, which involves making a master stamp with a nanostructure to transfer concaves on an aluminum substrate, which is then followed by anodization for regular pores. The last is conducted by sputtering the Al film onto a substrate, e.g., Si; this method can integrate AAO with different substrates for further applications.

The pore structure and profiles of AAO controlled by parameters, as mentioned in Figure 2 and Figure 3, play a pivotal role in various applications. Since the 1950s, thousands of AAO-related papers have been published in international journals, covering a wide range of applications. These include nanomaterial synthesis [7,29,30,31,32], electrical humidity sensors [33,34,35,36,37,38,39,40,41,42,43,44,45,46], optical sensors [47,48,49,50,51,52,53], photonic and electronic devices [54,55,56,57,58,59,60,61,62,63,64], photocatalysis [65,66,67], bio-applications [68,69,70], and other fields [71,72,73], as highlighted in Figure 1. A noteworthy contribution to AAO-based nanomaterial synthesis is the work proposed by Masuda et al. [74]. They demonstrated a two-step process that involves duplicating the nanostructure of AAO to synthesize nanoporous metal material for color applications. The material-and-structure-dependent AAO properties can be promoted by coating functional materials in pore structures and utilizing the pore geometry by depositing a metal layer for sensors and various applications. For instance, in 2000, Nahar et al. [34] proposed research on AAO humidity sensors and the adsorption mechanism on AAO pore walls. Their work allowed for the electrical measurement of ambient humidity using capacitance, achieved through the parallel capacitor structure. In terms of AAO optical sensors, the SERS application is the most well-known method for substance detection. It is also achieved by nanostructured metal layer deposition. The first SERS research by AAO substrate was proposed by Qiu et al. [75] in 2008. They directly deposited Ag on top of AAO to analyze its SERS phenomenon. The optical interference of AAO’s structure was proposed by Van Gils et al. [56]. The optical property of porous AAO was evidenced to change with structure modulation. The catalyst support AAO template [65,66,67] is generally by coating TiO_2_ particles in AAO nanopore structure or nanomaterial synthesis to fabricate TiO_2_ nanotubes. In industry, the characteristic of AAO is utilized on surface finishing for added value, increasing surface hardness and anti-corrosion for products. In brief, Figure 1 summarizes the overall interactive relation among the process parameters, nanoporous structure, and application, especially for sensors; those will be discussed in detail below in this review. 

### 1.1. Brief Development History of AAO

AAO is a well-known nanomaterial template, and its development has a long-term relationship with the rise of nanotechnology and nanomaterials. There are several important milestones in the development history of AAO. The first is the invented electron microscope by Max Knoll and Ernst Ruska in the 1930s, allowing people to examine the structure of matter below the micrometer level. In 1953, Keller et al. [76] proposed that AAO exhibits a hexagonal pore and cell model. From the current point of view, it is quite close, except for the pore shape. In 1970, O’Sullivan and Wood [77] proposed the anodization of a variety of acids to explain the formation mechanism of anodized alumina, allowing us to further understand the chemical reaction and AAO formation mechanism. In 1981, Thompson et al. [78] proposed the diffusion mechanism of anions and established a more complete theoretical basis for AAO formation. In 1995, Masuda et al. [74,79] proposed a two-step nanostructure duplication from AAO and proposed to synthesize nanoporous metal for color application. The paper was published in the Science Journal, which referred to the highly cited AAO fabrication and applications of AAO in the future, making AAO the most famous nanomaterial template. Until now, many teams have cited Masuda’s publications for two-step AAO preparation. In 2000, Nahar et al. [34] proposed the application of AAO as a humidity sensor and discussed the basic concept and water vapor adsorption mechanism. In 2006, Woo Lee et al. [25] developed a method of HA to grow AAO at high voltage. The growth rate is more than ten times faster than that of traditional mild anodization (MA) at a much lower voltage, and the bamboo structure produced by the MA–HA process was also proposed. Followed by the two-step anodization process, a pretextured Ni mold nanoimprinting method was also proposed by Masuda et al. [61] in 2006, which can leave not hexagonal but square or triangle arrays for special structure. In 2008, Bai et al. [80] used a fractional factorial design method to prepare ordered AAO with varied pore diameters in sulfuric acid at relatively high potentials and electrolyte concentrations. In the same year, the first SERS research concerning the AAO substrate was proposed by Qiu et al. [75] by directly depositing Ag on top of AAO to analyze its SERS phenomenon. In the next year, Sulka et al. proposed a two-step AAO fabrication process under a relatively high temperature with a discussion of the growth rate and pore geometry [22]. In 2011, Zaraska et al. [81] reported AAO membranes with varied pore diameters and thicknesses by adjusting the two-step anodizing duration and the post-pore widening time in phosphoric acid immersion. The effect of applied voltage, electrolyte type, and Al purity on growth rate was discussed. It is noted that the traditional AAO was fabricated with high-purity Al (≥99.99%) at low temperatures of −3~10 °C to diminish the Joule heating effect. In 2009 to 2014, Chung et al. [82,83,84,85,86] demonstrated the feasibility of nanoporous AAO fabrication from low-purity Al foil (≤99.5%) under 20–60 V at room temperature (RT), without cooling equipment, using HPA with a small negative potential followed by positive voltage, which could relax Joule heat to overcome the limitation of the conventional high-purity and low-temperature AAO process. In 2017, the evolution of widened pore diameter and characteristics of room-temperature AAO by different concentrations of 0.3–0.9 M oxalic acid for structure control was investigated by Chung et al. [87]; a new phenomenon was found and interpreted, where high oxalic acid concentration resulted in AAO walls containing more anions to further decrease etching rate during widening. In 2019, a thin-film spherical AAO template was reported using a superimposed 3D AAO-on-beads nano–micro structure [88] that was used for greatly enhancing sputtered thin-film TiO_2_ photocatalysis due to the large-surface-area substrate. In 2020, the AAO electrical sensor was further applied to gas detection by Podgolin et al. [89], which brought out more possibilities for the subsequent development of AAO. Also in 2020, Wang et al. [90] reported a bamboo-like AAO loaded with silver particles, which was used as one-dimension photonic crystals (PCs) to absorb light with a specific wavelength and enhance the local electric field as the SERS substrate. In 2021, Lim et al. [91] presented PCs based on the bamboo-like AAO modified with TiO_2_ to enhance the photocatalysis of methylene blue (MB). The photonic stop band (PSB) of bamboo-like AAO was tunable and helpful for harvesting the incident light efficiently. However, to form the periodic, long-range, ordered, bamboo-like AAO, a time-consuming fabrication process (10~72 h) is unavoidable [25,90,91]. Also in 2021, Gasco-Owens et al. [92] studied the growth mechanism of the in situ electrochemical large-pore anodizing of 5657 aluminum alloys with several phosphoric acid concentrations and voltages. In brief, the growth, morphology, and characteristics of nanoporous AAO and its wide applications have attracted great attention in traditional and advanced industries as well as in scientific research.

### 1.2. AAO Formation Mechanism

Nanoporous AAO is produced under a specific electrochemical reaction. It is mainly caused by the reaction of oxygen ions in the electrolyte with aluminum ions on the surface of the aluminum substrate to produce oxide [93]. The anode undergoes an oxidation reaction to form alumina, which can be explained by following five procedures:

(1) The aluminum metal located at the anode is affected by the applied voltage and dissociates to form aluminum ions, and the electric field drives the aluminum ions to move to the oxide layer:
  Al_(s)_ → Al^3+^(oxide) + 3e^−^ (1)
(2) A small number of water molecules dissociate into oxygen ions or hydroxide ions:
  H_2_O_(l)_ → 2H^+^_(aq)_ + O^2−^(oxide) (2)
  H_2_O_(l)_ → H^+^_(aq)_ + OH^−^(oxide) (3)
(3) Subsequently, oxygen ions or hydroxide ions are attracted to the positive potential of the anode and react with aluminum metal to form aluminum oxide, with the equations as follows:
  2Al^3+^_(aq)_ + 3O^2−^_(aq)_ → Al_2_O_3(s)_ (4)
  2Al^3+^_(aq)_ + 3OH^−^_(aq)_ → Al_2_O_3(s)_ + 3H^+^_(aq)_ (5)
(4) The hydrogen ions provided in the electrolyte and in the Equation (3) will dissolve aluminum oxide to form aluminum ions and water molecules:
  Al_2_O_3(s)_ + 6H^+^_(aq)_ → 2Al^3+^_(aq)_ + 3H_2_O_(l)_ (6)
(5) Hydrogen ions are also attracted by the cathode electric field, forming hydrogen gas at the cathode and electrons:
  2H^+^_(aq)_ + 2e^−^ _(aq)_ → H_2(g)_ (7)

When the dissolution is equal to formation, the reaction rates of Equations (6) and (7) will reach equilibrium. Therefore, all reactions of porous AAO can be classified into two procedures: anodization and electrochemical dissolution of the aluminum substrate—that is, Equations (4)–(6), which promote the dissolution and formation of aluminum oxide; the electric field assists the dissolution. When AAO grows, the pores will show a hexagonal crystal (cell) arrangement. This is because the volume of the alumina will expand; so, the volume expansion before and after the reaction will be affected by the anodic oxidation parameters and the pores will be affected by each other, which is called “self-organization”. The force of pushing each other creates a regular arrangement of hexagonal pore templates.

According to the description of the growth mechanism of AAO pore formation by Thompson [78] and Jessensky [94], when aluminum (Al) forms alumina, the volume will increase by more than 30% [94]. The internal stresses that interact with each other during the volume expansion process lead it to organize itself. Therefore, the AAO pores gradually reach the densest regular arrangement of the hexagons with the oxidation process. After the internal stress is densest, packing in the horizontal direction, AAO only grows in the vertical direction and forms a structure with a high aspect ratio.

**Figure 4:**
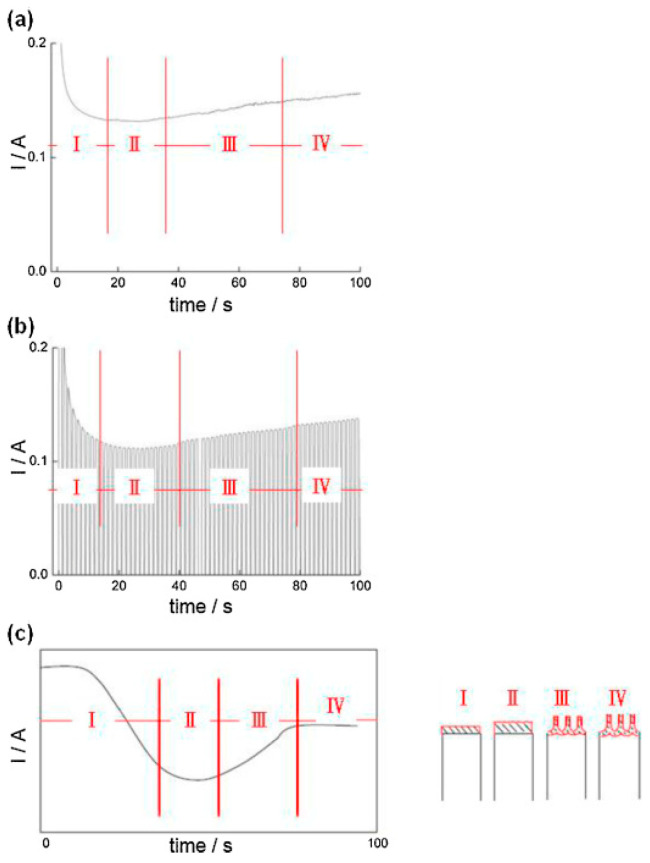
The four stages of the I-t current curve of the (**a**) DCA and (**b**) HPA. (**c**) The structure variation in four sections as periods I, II, III, and IV for various mechanisms. Reprinted with permission from Ref. [83]. Copyright 2011, Elsevier.

Figure 4a,b show the current–time (I-t) curves from anodization between the DCA and HPA processes. The different I-t curves are related to the distinct anodizing mechanism. The current always generates a high value with time during the DCA process that increases the Joule heating effect to accelerate dissolution. This is the reason that DCA is generally performed at a low temperature. However, the current of HPA at the negative period shows zero current so that Joule heat is significantly diminished with the negligible cathodic current and the electrolyte liquid for an effective cooling. Therefore, HPA is able to be performed with low-purity Al and a relatively high temperature of 25 °C. The global evolution of current with time for DCA and HPA is similar—that is, initially decreases and then gradually increases. The anodic oxidation process, whether carried out in DCA or HPA, can be divided into four stages, as shown in Figure 4c. Upon applying a specific potential, a barrier-type oxide layer will rapidly form on the surface of the aluminum substrate, known as the first stage (Stage I). Since the initial aluminum metal acts as a conductor, the current value during this stage starts high but drastically reduces as the oxide layer forms. In the second stage (Stage II), a dense oxide structure starts to form, with this stage having the thickest oxide layer and, consequently, the lowest current. By the onset of the third stage (Stage III), fully developed nanoscale pores begin to grow. At this point, the path through the bottom of the pores offers the least resistance to current flow, resulting in a concentration of current. The barrier layer is thinner than in the second stage, leading to an increasing current as AAO growth starts. In the final stage (Stage IV), a balance between the dissolution rate of the pores and the oxidation rate is achieved, causing the oxide layer to gradually thicken. After a certain duration of growth, stress leads to the rearrangement of pores into a highly regular pattern, forming ordered pore structures in the anodized alumina, which is known as “self-organization”.

### 1.3. Controllable Parameters of AAO

AAO is a nanomaterial template formed under specific electrochemical conditions; so, the adjustable parameters include purity, voltage, temperature, and electrolyte concentration. In the traditional processes, the low-temperature (−5~15 °C) anodization experiment is easier to obtain complete and regular AAO pores, because the low temperature can slow down the chemical dissolution rate of the interface between the oxide layer and the electrolyte. In a low-temperature environment, the Joule heating effect generated by the electric-field-assisted dissolution at the bottom of the alumina pore is taken away and the structure is prevented from rupturing. Therefore, some scholars began to focus on how to make the ambient temperature reach a lower temperature in order to obtain a better pore arrangement, such as adding alcohol to the electrolyte to reduce the freezing point of the electrolyte to below 0 °C. However, the low-temperature environment will suppress the growth rate of AAO and reduce the current density during the reaction, which directly affects the thickness of the AAO layer. In addition, the cost and energy consumption of the ice water machine also indirectly increases the manufacturing threshold of AAO; so, many studies hope to increase the reaction temperature of AAO to 20–30 °C. The detailed anodization processes under low to relatively high temperatures will be discussed in Section 2 and Section 3.

**Figure 5:**
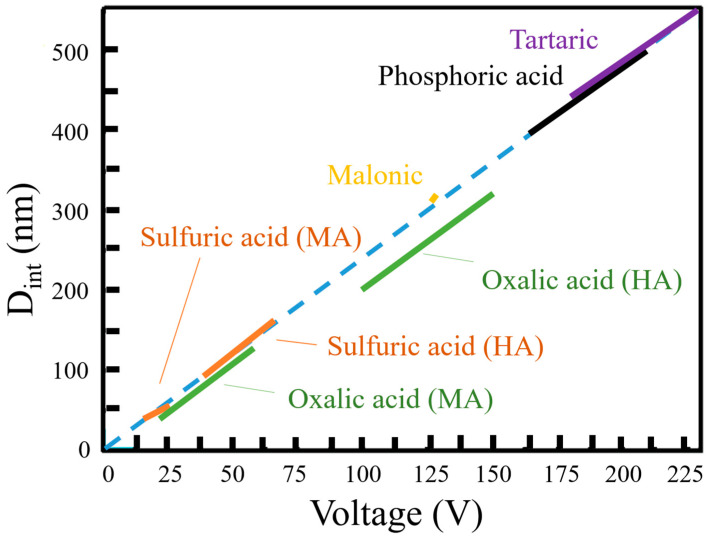
The anodization voltage vs. D_int_ plot and the commonly used electrolytes.

Anodization potential can be categorized into MA and HA. For AAO processes, a lower applied potential cannot produce regularly arranged pores, while a higher potential will accumulate excessive Joule heating effect due to the accumulation of electric charges, which causes the current density to be too high and destroys the nanostructure. Therefore, choosing the appropriate potential for different electrolytes can stably prepare regular pore morphology. The potential of the MA is the traditional low-temperature DCA process. However, due to the different dissociation degree of different electrolytes, the applied anodic potential will also change. Among the common electrolytes, the potential used for sulfuric acid is 20–25 V, oxalic acid is 30–40 V, and phosphoric acid is 160–195 V [23,24]. Sulfuric acid has the lowest pH value and the highest dissociation degree; so, the suitable potential is the lowest, followed by oxalic acid and phosphoric acid. Recently, the organic acids such as malonic acid, tartaric acid, etc., are generally required to anodize at a higher voltage because of the low dissociation degree [23,24]. The relationship between the voltage and interpore distance (D_int_) is shown in Figure 5 by proper anodization voltage to each electrolyte. The D_int_ is directly proportional to the applied voltage with a relationship of about 2.5 nm/V. HA is proposed by W. Lee et al. [25]; by applying a higher voltage than MA, a higher oxidation reaction rate can be achieved, and AAO can be prepared more efficiently. Among them, the applicable potential of sulfuric acid is 40–70 V, and that of oxalic acid is 100–150 V, which has encouraged more scholars to study in the direction of improving the efficiency of high-potential anodic oxidation. However, a fast anodizing reaction is accompanied by a larger current, so the heat accumulation is more obvious, making the pore easier to collapse and burn. The level of applied voltage has a great relationship with the pore diameter (D_p_) and D_int_. The applied potential is proportional to D_int_ (Figure 5) and has a positive relationship with D_p_.

Another important parameter during the anodization process is electrolyte concentration. In 1981, Thompson and Wood [42] proposed that in different acidic electrolytes, anions will remain in the AAO pore wall, especially sulfuric acid, which is followed by oxalic acid, phosphoric acid, and chromic acid, which leads to almost no contamination of the AAO pores. Sulfuric acid, oxalic acid, and phosphoric acid were the most commonly used electrolytes for the preparation of AAO, while chromic acid was not considered for use because of concerns about heavy metal pollution. In addition, the concentration of the electrolyte will directly affect the dissociation ability. If more anions participate in the reaction, the growth rate will increase and the anion concentration in the pore will increase. Different electrolytes need to arrange an appropriate anodic potential according to the acid dissociation degree, while the lower dissociation degree requires a higher potential, such as phosphoric acid and some weak organic acids. Furthermore, higher concentration provides a high conductivity of electrolytes and results in fast growth of AAO.

**Figure 6:**
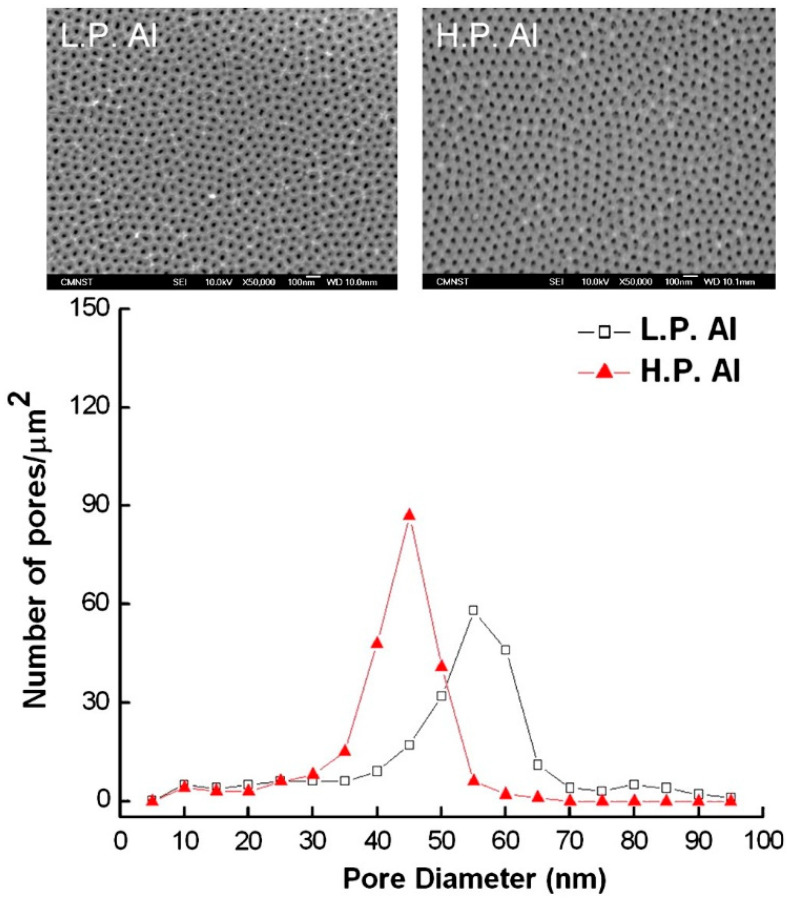
The SEM images and pore distribution of AAO prepared from high-purity and low-purity aluminum under HPA at 30 V and 25 °C. Reprinted with permission from Ref. [86]. Copyright 2011, Elsevier.

The purity of the aluminum substrate influences the regularity and arrangement of AAO pores. Figure 6 shows that a better pore structure is formed by anodizing high-purity than low-purity aluminum, as well as the more obvious unimodal pore size distribution. So, many researches of AAO are still using two-step anodization of high-purity aluminum. In order to further reduce costs or integrate with industrial manufacturing lines, the low-purity aluminum or alloys rather than traditional high-purity aluminum are investigated by some researchers. But they are facing the problem of poor regularity of low-purity aluminum as the substrate for potential application.

### 1.4. AAO-Based Sensor Applications

AAO applications include nanomaterial synthesis, electrical sensors, optical sensors, photonic and electronic device, photocatalysis, hardness or anti-corrosion improvement, and other categories. With the rapid development of micro–nano technology and automated industry, sensor-related issues have received more attention. AAO-based sensors can be divided into two categories: electrical sensors and optical sensors. The electrical AAO-based sensor is primarily based on two types of capacitance and resistance, usually depositing a thin metal film on the surface of AAO as an electrode for humidity [34,35,36,37,38,39,40,41,42,43,44] and gas [89] measurement. Many more publications on pure AAO humidity sensors than gas ones are probably attributed to their high response in sensing relative humidity for environmental and industrial applications.

Humidity detection is crucial in modern industries and technology sectors, encompassing fields such as weather forecasting, agriculture, air conditioning, food, and artwork preservation. Various materials including ceramic [95,96], semiconductor [97,98], polymer [99], and carbon-based materials [100,101] are used for relative humidity (RH) sensing. As one of the ceramic materials, AAO-based sensors possess good thermal stability, anti-corrosion, high mechanical strength, and large sensitivity under high-RH conditions. Moreover, the humidity sensors can be based on various types of capacitive, resistive, mass-sensitive, and electromagnetic features, and both the capacitive and resistive types are popular and suitable for commercial use. The traditional AAO humidity sensor was generally produced by two-step anodization from small-area and high-purity (>99.99%) aluminum, which is a high-cost and time-consuming process. Low-cost processes using low-purity (<99.5%) aluminum is a potential direction for practical application. Furthermore, the performance of humidity sensors can be evaluated by two main indicators. One is response or sensitivity, which distinguishes different humidity levels. The other is the response–recovery time, which indicates the reaction speed of the sensor in adapting to changing environmental conditions and is defined as reaching a stable value with less than 10% error under cyclic humidity changes. The different AAO-based sensor mechanisms based on anions in the pore wall, magnetic field presence, and pore geometry will be discussed in Section 4.1 to meet the requirements of high response– and short response–recovery time.

In terms of the AAO optical sensor, there are four kinds of mechanisms, i.e., reflectometric interference spectroscopy (RIfS), photoluminescence (PL), surface plasmon resonance (SPR), and surface-enhanced Raman scattering (SERS). The RIfS formed by the interference of the reflected light of the film is used to detect the analyte through the change in the effective optical thickness [102,103,104,105]. The PL spectrum of the analyte or the fluorogenic dye is changed after the analyte is combined with the AAO-based sensor [106,107,108,109]. SPR is the oscillation of electrons at a metallic surface stimulated by an incident light. The analyte is detected based on the changes in the absorbance of SPR of the substrate [110,111,112,113,114]. Recently, SERS based on AAO is the most-published topic and can be applied for trace detection of various substances, which is a technique of enhancing the Raman signals by the localized strong electrical field known as “hot spots” [115,116,117,118,119,120,121]. AAO can be a solid support for the nanoparticles [115,116,117] because of its high specific surface area, tunable pore diameter, and good biocompatibility [115,116,117]. In addition, AAO can be a temperature template for fabrication nanorods [118] or nanowire [90] arrays. Moreover, bamboo-like AAO can also be used as one-dimension photonic crystals (PCs) for SERS sensing [90]. At last, the peripheral, the gap, and the tips of the pores coated with metal film causing plasmonic oscillation using irregular AAO was demonstrated [120,121]. For the AAO-based optical sensors, our comprehensive review of four different mechanisms is focused on SERS applications because of the excellent performance and wide applicability. The recently reported mechanisms and applications using AAO to fabricate the SERS substrate are clarified in Section 4.2. The high-performance sensors will play a crucial role in the Internet of Things in our living environment and promote our quality of life in the future.

## 2. Advancement of AAO Template and Its Geometry at Low Temperature Process

In traditional technology, AAO is manufactured at a low temperature of −5~15 °C from high-purity aluminum substrate by applying a fixed voltage or current in a specific electrolyte concentration. However, in recent years, with the development of nanotechnology, different processes or methods have been developed to improve the results and applications. AAO fabrication processes can be divided into three categories: The first is the well-known two-step process proposed by Masuda et al. [74] and introduced in Section 2.1. Until now, many teams still use this method to prepare AAO. Another is the pretexturing process and stamp nanoimprinting method [122,123] described in Section 2.2. By making a master stamp with a nanostructure to transfer concavity to the aluminum substrate, the surface of the patterned aluminum foil can be obtained; then, followed by anodization, AAO is formed with regular pores due to the effect of the electric field concentration. The other process is conducted by sputtering Al to a substrate material e.g., Si [124,125,126,127] (Section 2.3). This method can integrate AAO with different substrates for further application. However, due to the disadvantages of high cost, high time-consumption, and limited area for preparing nanotemplates, the hope is that there will be cheaper and more convenient and manufacturing processes to improve these methods in the future. Therefore, growing AAO directly from aluminum substrates is still the mainstream method today. To improve the efficiency of the AAO process, the hard anodization and one-step anodization method have become more popular and are discussed in Section 2.4 and Section 2.5, respectively.

### 2.1. Conventional Two-Step Process

**Figure 7:**
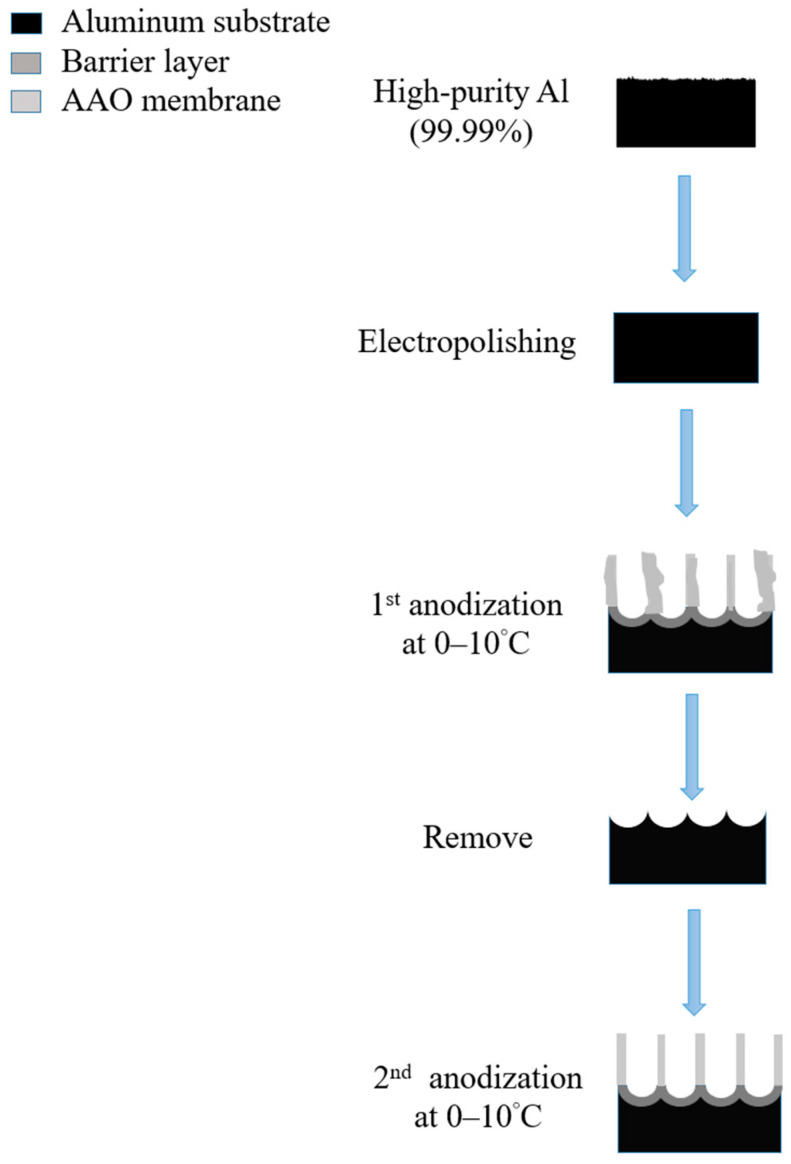
The schematic diagram of 2-step anodization process. First, the sample surface is cleaned, followed by electrochemical polishing to make the aluminum surface smooth. In first step of AAO growth, it usually forms uneven structures. Therefore, chemical etching is used to remove them before growing the second-step regular AAO.

Masuda et al., in 1995 [74], proposed the two-step anodization method for AAO technology, as shown in Figure 7, which laid the foundation for the AAO processes and applications. Many groups still follow this approach [128,129,130,131] today for AAO-related research. The first AAO can leave a pre-pattern after the removal process, making the hexagonal structure more regular during the second step of anodization. Two-step anodized alumina using high-purity aluminum substrate can obtain a highly ordered nanopore structure. However, it also brings high cost, long processing time, and other shortcomings. The price of high-purity aluminum is more than 1000 times that of commercial low-purity aluminum, which increases the cost of experiments. The two-step manufacturing method makes the whole process more complicated. The first AAO growth and removal step usually takes more than 6–10 h. In order to reduce the time required, some publications in recent years used the one-step process to prepare AAO for different applications. For example, Li et al. reported the effect of hydrothermal treatment on porous anodic alumina generated by one-step anodization [132]. Yu and Chung used one-step anodization and pore widening to produce irregular pores for high SERS enhancement with the assistance of the hybrid electric-field-enhanced plasmonic mechanism [120,121].

### 2.2. Pretexturing Process

**Figure 8:**
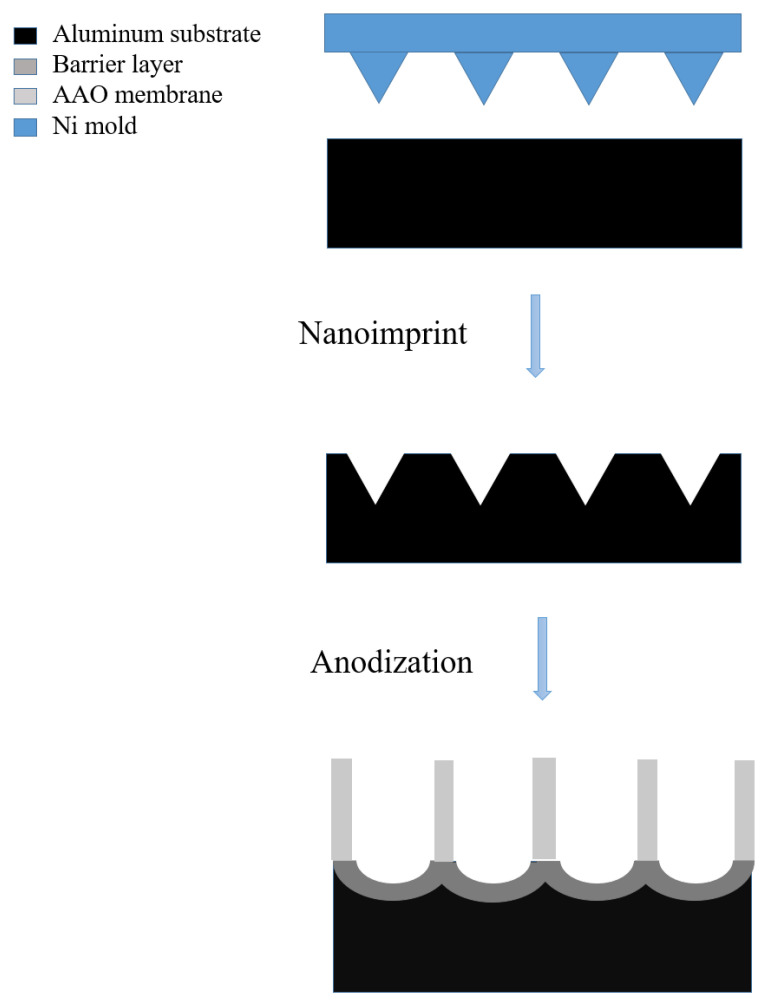
The schematic diagram of stamp nanoimprinting process to fabricate ordered AAO structure.

Figure 8 shows a typical schematic diagram of the pretextured stamp nanoimprinting method [61,63,124,125]. The imprinted concaves replace the pre-positioning of the first anodization and guild to create a long-range ordered AAO nanostructure. This method uses a Ni mold to leave concave patterns on the aluminum substrate. The advantage is that the stamp-imprinting method is able to fabricate a AAO nanopore array more than several mm in size and make it possible to create not hexagonal but square or triangle arrays for special structure [133]. However, this method also has the disadvantage of high cost and difficulty in Ni mold preparation, so it is still not popular in AAO fabrication.

### 2.3. AAO on a Si Substrate

**Figure 9:**
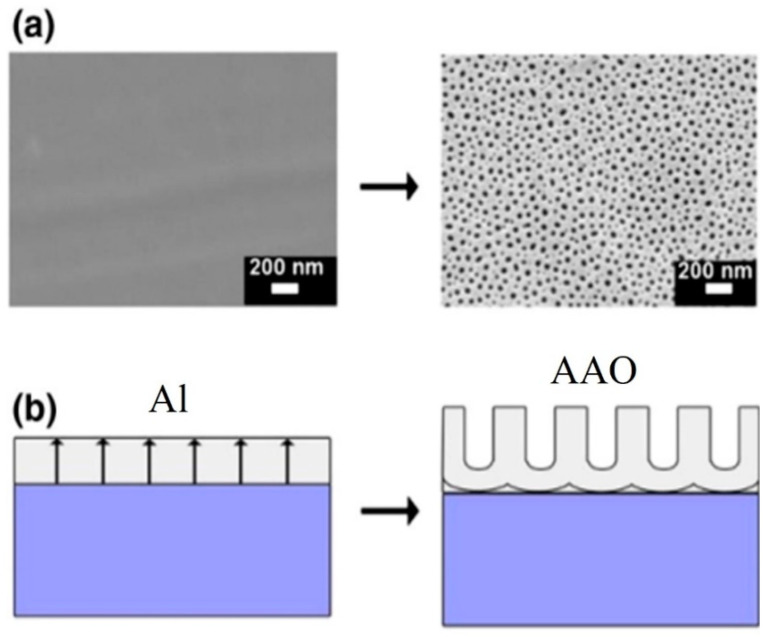
(**a**) The SEM images and (**b**) the schematic diagram of AAO on the Si substrate process from the IBS-deposited smooth Al film.

In order to grow AAO on different substrates such as silicon wafers or glass for various applications, the deposition of aluminum thin films and subsequent anodization are critical problems to study [134,135]. However, the hillocks that form during sputtering on heterogeneous substrates have been a challenging issue [136,137,138]. Moreover, traditional processes with two-step anodization on Si wafer [134,135] increase the complexity and prolong processing time. The thick AAO on Si substrate is usually produced by anodizing the e-beam-deposited thick Al film rather than the sputtered thin Al film. The uniformity of Al film is important for AAO pore quality. The ion beam sputtering (IBS) in the high-vacuum ambient condition is beneficial for the quality of the Al film. Figure 9 shows the schematic diagram of AAO on a Si substrate with anodizing the IBS Al film [127]. Figure 9a is the smooth Al film deposited by ion beam sputtering (IBS) in ultra-high vacuum and its porous structure after HPA anodization. The anodization process is performed at 40/−2 V under 15 °C. The very smooth surface morphology without any particles is attributed to the vacuum condition. In this scenario, the prepared AAO exhibits results similar to the one-stage anodization of bulk aluminum substrates, with no occurrence of hillock structures. The distribution of pore size is good, with an average pore size of 31.06 ± 6.34 nm even in a short processing time. The AAO formation from the sputtered Al thin films on Si is expressed by the schematic diagrams in Figure 9b. The electric field direction is indicated by arrows. The electric field during anodization under applied potential difference is uniformly distributed on the surface of the flat and smooth Al film, neglecting the edge effect. This method has potential for integrating AAO on various substrates for applications in different fields.

### 2.4. Hard Anodization Process

Anodization potential can be categorized into MA at a low potential and HA at a high one. For the AAO process, a lower applied potential is not good for producing regularly arranged pores, while a higher potential will accumulate an excessive Joule heating effect due to the accumulation of electric charges, which causes the nanostructure to be destroyed. The respective potentials of the MA and HA processes in electrolyte acid are drawn in Figure 5. Generally, the voltage of the HA process is only discussed based on sulfuric acid and oxalic acid, which have a lower MA voltage. Due to the different dissociation degree of electrolytes, the applied potential will also change. Among the common electrolytes, the MA potential used for sulfuric acid is 20–25 V, for oxalic acid is 30–40 V, and for phosphoric acid is 160–195 V. The suitable potential of sulfuric acid is the lowest because it has the lowest pH value and the highest dissociation degree, followed by oxalic acid and phosphoric acid. In addition, W. Lee et al. [25] proposed the HA process by applying a greater voltage than MA to achieve fast and efficient AAO fabrication at a relatively low temperature. The applicable HA potential of sulfuric acid is 40–70 V and that of oxalic acid is 100–150 V. However, a fast anodizing reaction is accompanied by a larger current, and the greater heat accumulation makes the pore easier to collapse and burn. So, the HA process is always conducted at a lower temperature of −5~10 °C [14,15,16,17,18,19,20,21,22,23,24,25,26,27,28].

**Figure 10:**
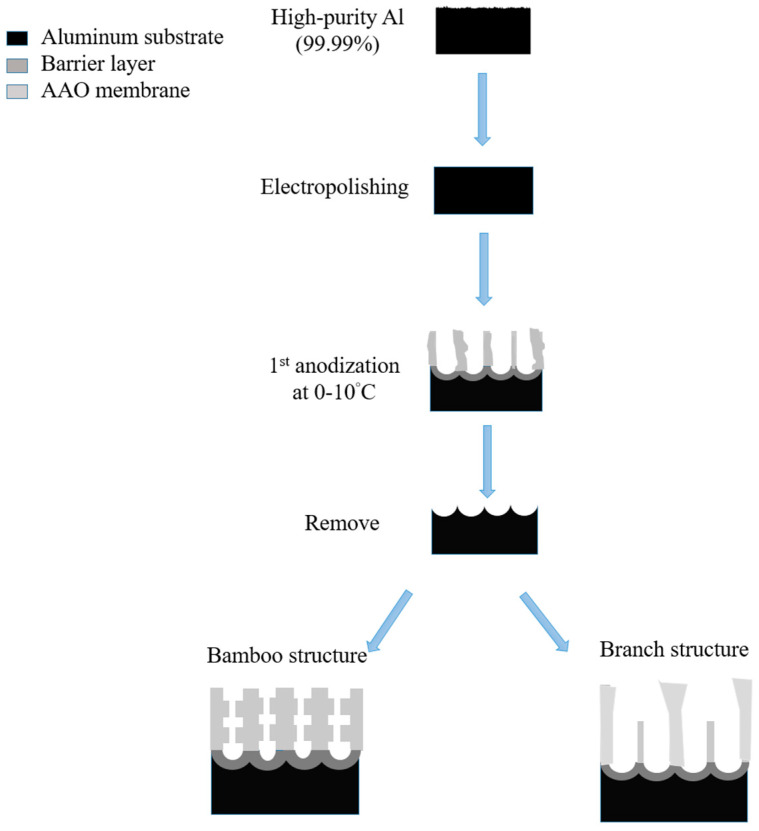
The special structure AAO fabrication process of bamboo structure and branch structure.

The HA process also opens the preparation of special structure AAO; the schematic diagram is drawn in Figure 10. For example, the development of the bamboo-shaped structure is the result of the alternating implementation of MA and HA voltages. The HA voltage would lead to a larger D_p_ and the MA voltage would result in a smaller D_p_. For several cycles, the AAO profile will grow in an obvious bamboo shape. On the other hand, the preparation of branched pores is achieved by a larger potential and followed by the same or reduced voltage. Due to the different electric field concentrations, the top view of AAO is diverged as a branch shape. The second potential will form smaller pores at the bottom of the original pores. However, it is a pity that there is quite little effective applications for special structures at present, and future research and discussion are still needed.

### 2.5. One-Step Low-Temperature Process

The traditional AAO fabrication process is primarily based on the two-step anodization method proposed by Masuda and Fukuda [74], which has gained widespread usage due to its well-ordered AAO nanostructure. However, this method brings high cost, long processing time, and other shortcomings; so, some groups studied the one-step anodization method at 0~10 °C to promote AAO fabrication efficiency and pore circularity together with some application of filtration [139] or optical sensors [140].

**Figure 11:**
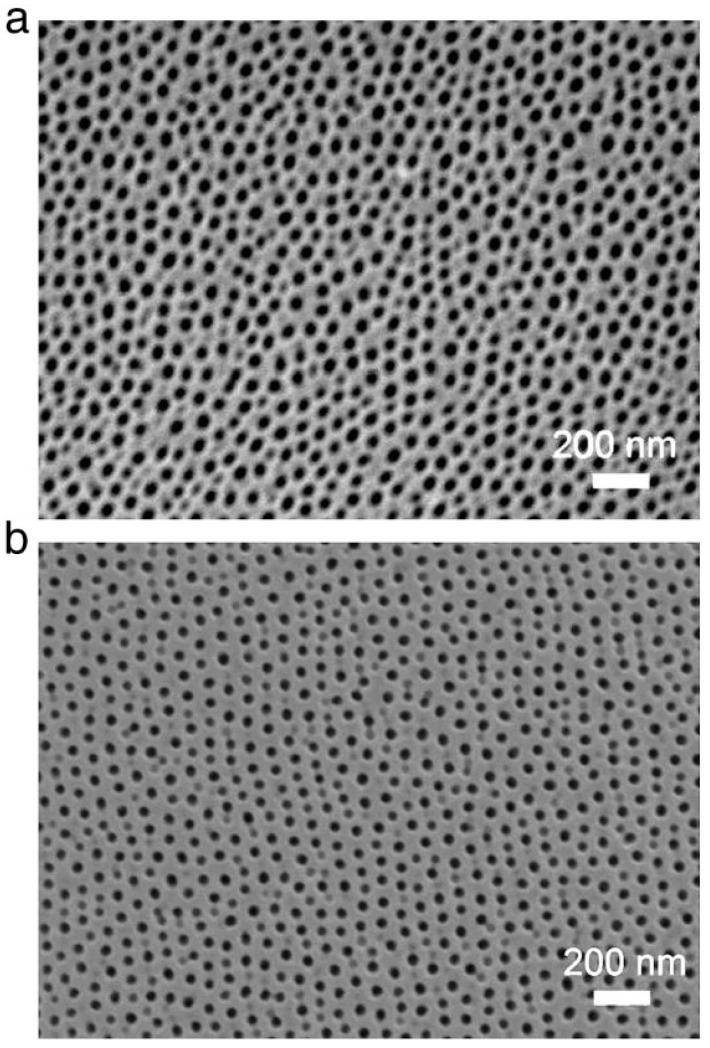
SEM micrographs of one-step AAOs fabricated by 0.5 M oxalic acid at 5 °C for 1 h with pore-widening process in (**a**) DCA mold and (**b**) HPA mold. Reprinted with permission from Ref. [85]. Copyright 2011, Elsevier.

Chung et al. proposed the one-step AAO fabrication process using DCA and HPA on high-purity aluminum foil at 5 °C for 1 h, followed by a 30 min pore-widening process in 5 wt% phosphoric acid, as shown in Figure 11a,b, respectively [85]. In both cases, the nanopores of AAO are clearly visible, but the HPA AAO exhibits more uniformity, higher circularity, and a more orderly arrangement compared with the DCA AAO. ImageJ analysis revealed an average pore diameter of approximately 40 ± 5 nm for DCA AAO films and 35 ± 5 nm for HPA AAO films. Due to the dissolution from the Joule heating effect, the pore diameter of DCA AAO is larger than that of HPA AAO. The continuously accumulated Joule heat in DCA is much higher than in HPA, with a cooling effect based on the duration of small negative voltage for negligible current. So, the larger pore size of DCA AAO over HPA is concerned with higher dissolution rate. Also, the 35 ± 5 nm pores of HPA AAO are around 92.6% compared with the 40 ± 5 nm pores of DCA AAO at only 82.9%. This indicates that the HPA process benefits the better uniformity of pores distribution with a low Joule heating effect.

**Figure 12:**
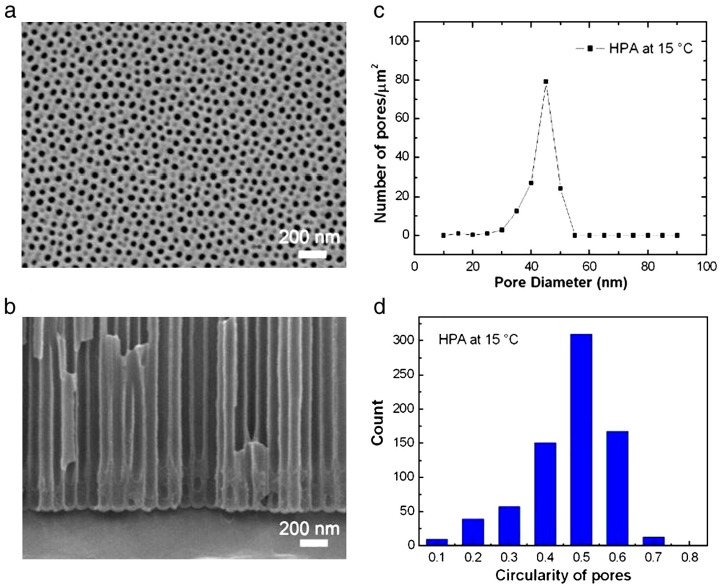
(**a**) SEM micrograph, (**b**) pore diameter distribution, (**c**) SEM cross-section view, and (**d**) circularity by ImageJ analysis. AAO is prepared by HPA from HP-Al in 0.5 M oxalic acid at 15 °C for 1 h. Reprinted with permission from Ref. [85]. Copyright 2011, Elsevier.

Figure 12a shows the top-view SEM micrograph of the one-step HPA AAO formed from high-purity Al foil at a higher temperature of 15 °C for 1 h followed by a 30 min pore widening process in 5 wt% phosphoric acid. The pores are still good at such a temperature. It is noted that the high potential DCA results in higher current density with defects in aluminum foil and destroys the balance of formation and dissolution rate as well as the electric breakdown, ruining the AAO nanostructure. Therefore, conventional AAOs primarily perform at a low temperature of −5~10 °C depending on the voltage magnitude. A sufficient cooling effect in HPA may diminish this ruined phenomenon. Figure 12b shows the cross-sectional SEM micrograph of the barrier layer of one-step HPA AAO in Figure 12a. HPA is beneficial for the one-step AAO at a relatively high temperature. The ImageJ software analyzed pore distribution and circularity in Figure 12a and is drawn in Figure 12c. The main pore diameter of AAO is 45 ± 5 nm, around 88.6%. The mean pore size of AAO increases with the elevated temperature but the distribution uniformity decreases compared with that in Figure 11 because of the temperature-enhanced dissolution to widen the pore and reduce the distribution uniformity. Figure 12d shows that the main pore circularity of one-step HPA AAO is from 0.5 to 0.6 higher than that of DCA at 5 °C.

## 3. Advancement of AAO Fabrication at a Relatively High Temperature

Traditionally, the fabrication of anodic aluminum oxide (AAO) involves a two-step process at low temperatures. In comparison with one-step AAO, the two-step process results in more regular pore arrangement and hexagonal structures. However, the low temperature of −5~15 °C suppresses the thermal effect while also slowing down reaction rates. This has led many researchers to explore elevating the AAO reaction temperature to relatively high temperatures [22,81,82,83,84,85,120,121,140]. Nevertheless, increasing the temperature generates more Joule heat, which may cause random defects in the AAO nanostructures. Many researchers tend to focus on the improvement of growth rate, but detailed discussions regarding the pore morphology and distribution are less emphasized. As mentioned, the HPA process with an effective cooling effect could reduce the resistive heating effect during anodizing and form adjustable nanopores at 20–30 °C, which will be discussed in Section 3.1. In oxalic-acid anodization, the commonly applied voltage is 40 V. while the relative high voltage of 50–60 V can be achieved by the HPA process. This helps to further improve the efficiency of AAO production and achieve the varied nanostructure. In addition, the pore widening process contributes to improving the circularity of the pores. We will explain and explore the pore-widening mechanism in Section 3.2. In Section 3.3, the one-step anodization method at room temperature will be introduced and compared with the two-step process in Section 3.1.

### 3.1. HPA at Relatively High Temperatures and Voltages

**Figure 13:**
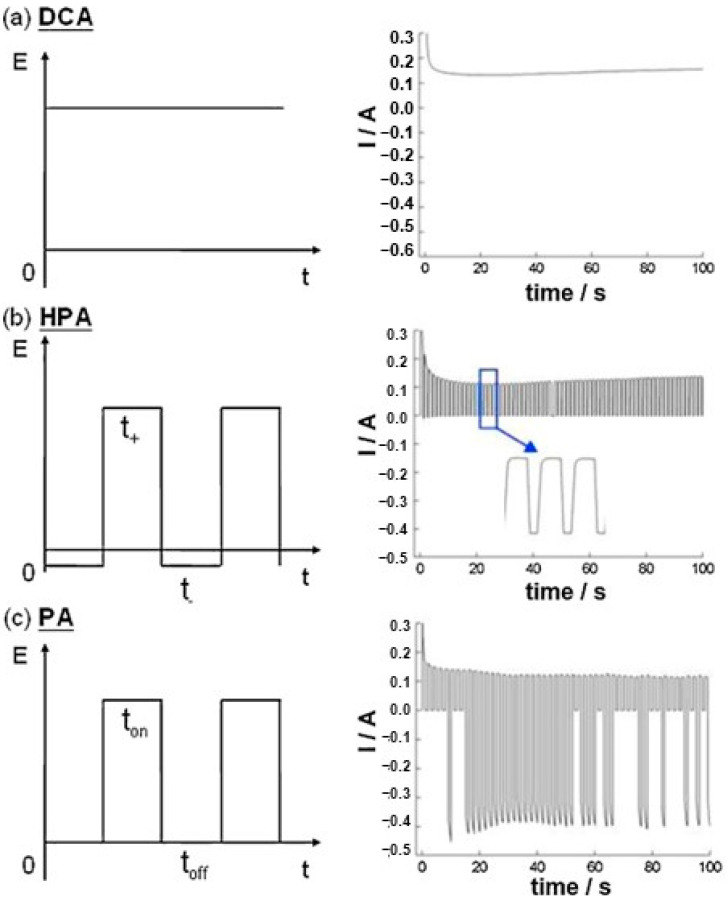
Comparison of the schematic applied voltage (V) and the measured current (I) as a function of time (t) in three kinds of voltage modes: (**a**) DCA, (**b**) HPA, and (**c**) PA. Reprinted with permission from Ref. [83]. Copyright 2011, Elsevier.

The voltage mode is an important parameter affecting the synthesis of AAO at relative high temperatures and voltages. Figure 13a–c show comparisons of the applied voltage (V) and the real-time measured current (I) as a function of time (t) in three kinds of voltage modes of DCA, HPA, and PA, respectively. The heat accumulation occurs in the continuous current–time diagram in DCA (Figure 13a), resulting in the thermally enhanced Joule heating effect. On the contrary, the effective cooling of HPA with a negligible current at small negative potential (Figure 13b) for the duration of t_-_ to diminish the dissolution effect benefits the formation of ordered AAO nanostructures at temperatures of 20–30 °C. Compared with conventional pulse anodization (PA), shown in Figure 13c, the distinct negative current is observed during PA for the duration of t_off_. It is like a capacitor discharging the charges accumulated for the duration of t_on_, so the current flow is opposite to form the cathodic current. The irregular reverse current is linked to the impurities of the substrate to make a rapid polarization change between the anode and cathode electrodes in a short time. For the duration of t_−_ of HPA, it can diminish the discharging effect with a small value of −2 V due to having insufficient potential for a cathodic reduction reaction. Accordingly, the cathodic current is nearly zero current.

**Figure 14:**
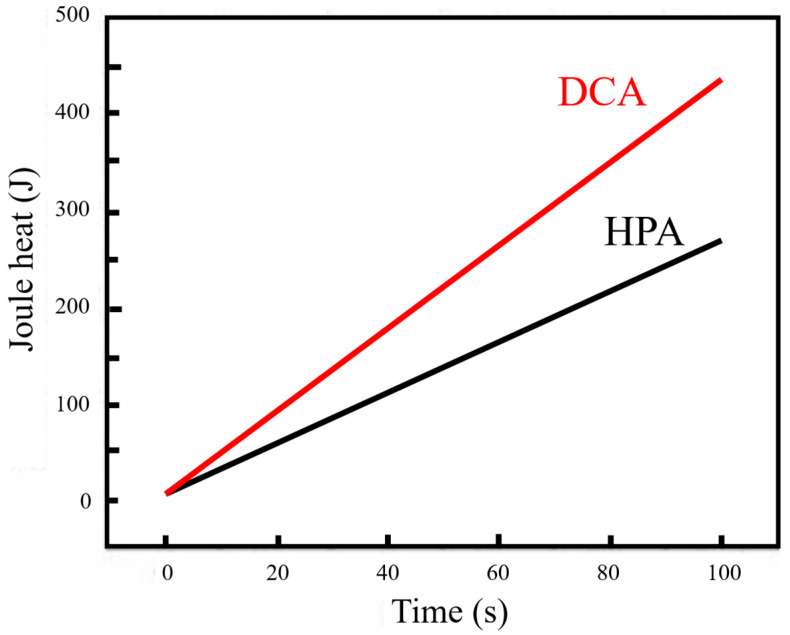
The comparison of Joule heating effect from DCA and HPA in the first 100 s of the anodization process.

The power generated during anodizing is concerned with the Joule heating effect and expressed as *P_joule_* = *I*^2^*R*, where *P_joule_* denotes watts, *I* denotes amperes, and *R* denotes ohms. The resistance (*R* = *ρL*/*A*) depends on the AAO’s structure of thickness (*L*), reaction area (*A*), and resistivity (*ρ*). However, the resistance of the electrolyte and Al-AAO template is difficult to accurately calculate or measure. Therefore, calculating the total energy consumed within a specific time interval provides a viable alternative approach by the formulae *P_joule_* (*t*) = *∫I*(*t*) *V dt* or *P_joule_* (*t*) = *I*(*t*)*·V*. The Joule heat generated by DCA and HPA during anodization for the first 100 s was computed using MATLAB R2008a V.7.6 and is shown in Figure 14. This evidences that a lower Joule heating effect for AAO with effective cooling mechanisms from HPA diminishes the dissolution reactions for better structure formation.

**Figure 15:**
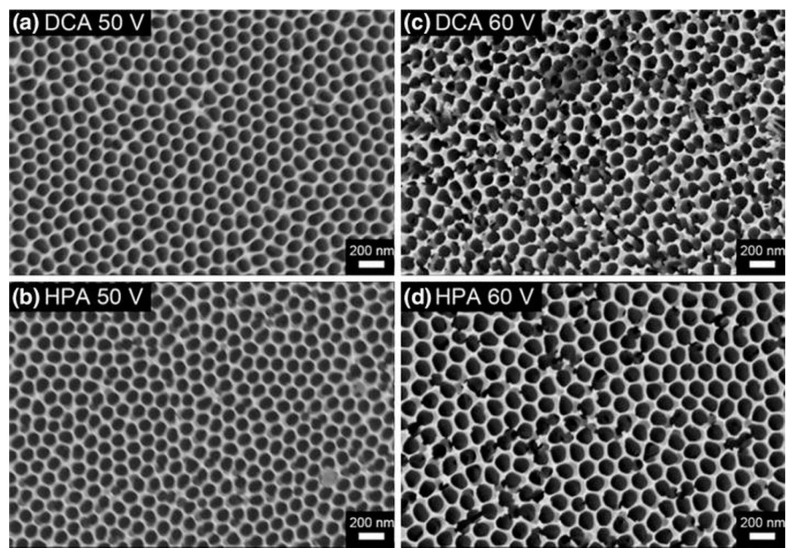
The SEM images of AAO nanopore structures fabricated by (**a**) DCA 50 V, (**b**) HPA 50 V, (**c**) DCA 60 V, and (**d**) DCA 60 V. Reprinted with permission from Ref. [86]. Copyright 2014, Microsystem Technologies.

**Figure 16:**
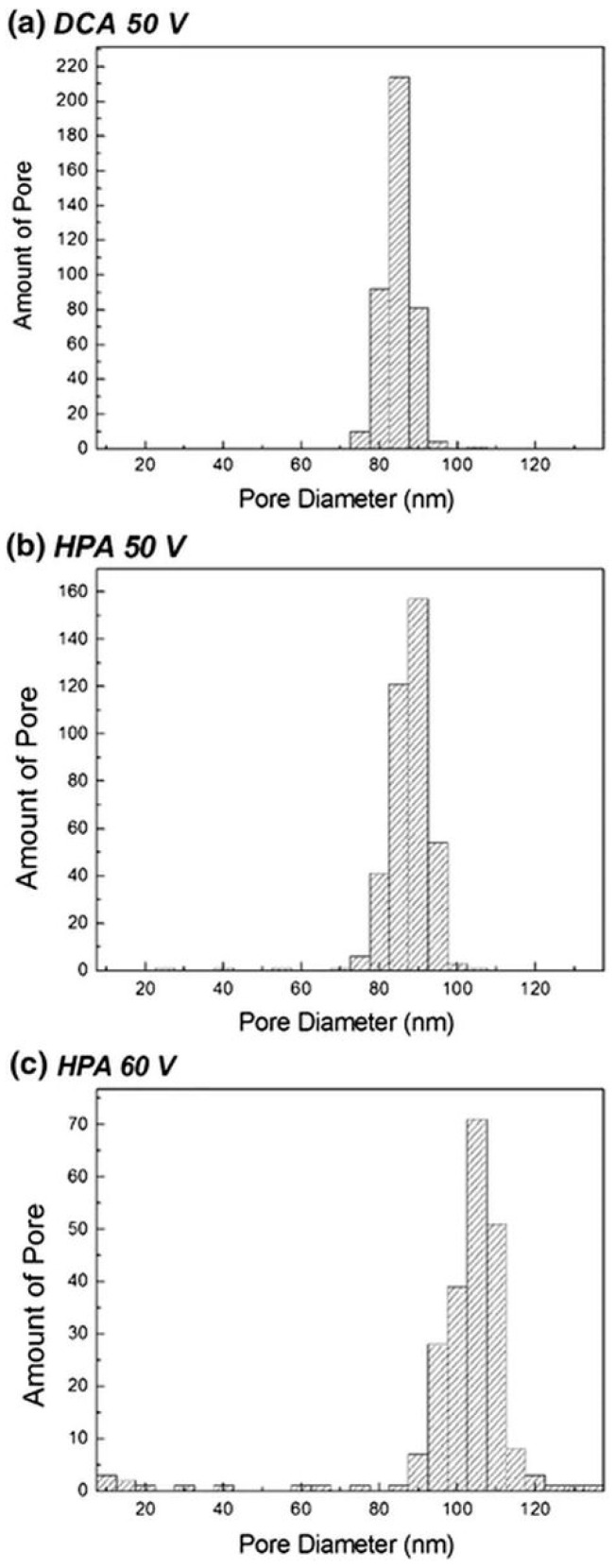
(**a**–**c**) The AAO pore distributions corresponding to Figure 15a,c,d, respectively. Reprinted with permission from Ref. [86]. Copyright 2014, Microsystem Technologies.

The SEM top views of AAO synthesized by DCA and HPA at 50 and 60 V in oxalic acid at 25 °C with the pore-widening process in 5 wt% phosphoric acid for 25 min are shown in Figure 15. The morphologies and pores of AAOs obtained using both DCA and HPA at 50 V (Figure 15a,b) remain complete, which is attributed to the accumulated heat not dissolving and the pore widening not over-etching the pore wall. However, at 60 V in (c) and (d), the AAO structure produced by DCA is apparently damaged in certain regions, and that by HPA retains a better pore structure due to the cooling effect of HPA resulting less dissolution in the original formation. The AAO pore wall is destroyed by the pore-widening process in DCA (Figure 15b). On the other hand, the AAO pore structure produced by HPA is relatively complete (Figure 15d). In addition, SEM images reveal that the distribution of pore size increases with potential, as plotted in Figure 16. For 50 V anodization, the pores occupy the main distribution of 85 ± 5 nm in DCA compared with 90 ± 5 nm in the HPA process. For 60 V, the range of AAO pore size by HPA increases to 105 ± 10 nm. It is noted that the D_p_ of the AAOs prepared at both anodization voltages are larger than those in low-temperature anodization due to the high-temperature (25 °C) anodization, followed by the pore-widening process.

### 3.2. AAO Pore Widening and Mechanism at 25 °C

**Figure 17:**
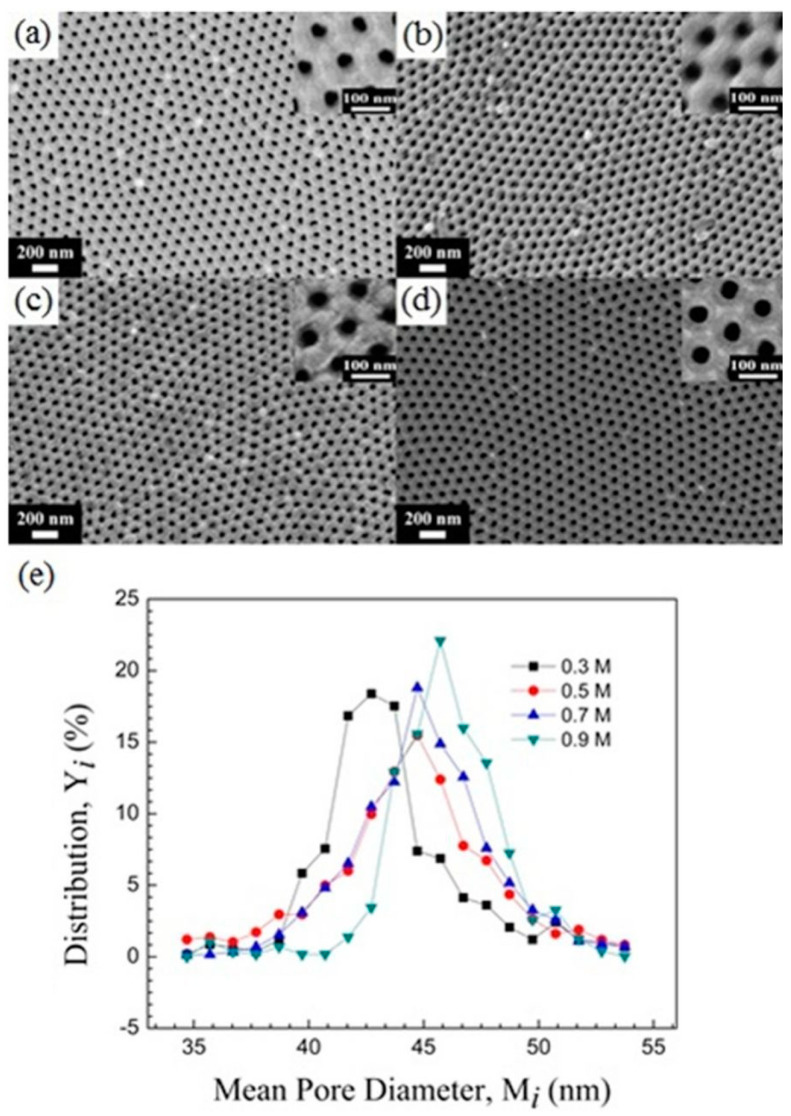
SEM images of AAO formed by HPA from HP-Al in (**a**) 0.3 M, (**b**) 0.5 M, (**c**) 0.7 M, and (**d**) 0.9 M oxalic acid at 25 °C for 2 h. (**e**) The AAO pore size distribution calculated by (**a**–**d**). Reprinted with permission from Ref. [87]. Copyright 2017, Journal of The Electrochemical Society.

Figure 17 depicts the SEM images of high-purity aluminum fabricated by HPA for 2 h in oxalic acid solutions with concentrations ranging from 0.3 M to 0.9 M at 25 °C. Although (a)–(d) exhibit similar results, the pore distribution plot in (e) reveals an increasing trend in AAO pore diameter with rising oxalic acid concentrations. The D_p_ values are 42.8 nm (0.3 M), 45.2 nm (0.5 M), 45.9 nm (0.7 M), and 46.5 nm (0.9 M), respectively. These variations are attributed to the oxalic acid concentration; as the concentration increases, more ions dissociate in the electrolyte, leading to higher current and enhanced dissolution in the chemical reaction, thereby causing an enlargement in AAO pore size.

**Figure 18:**
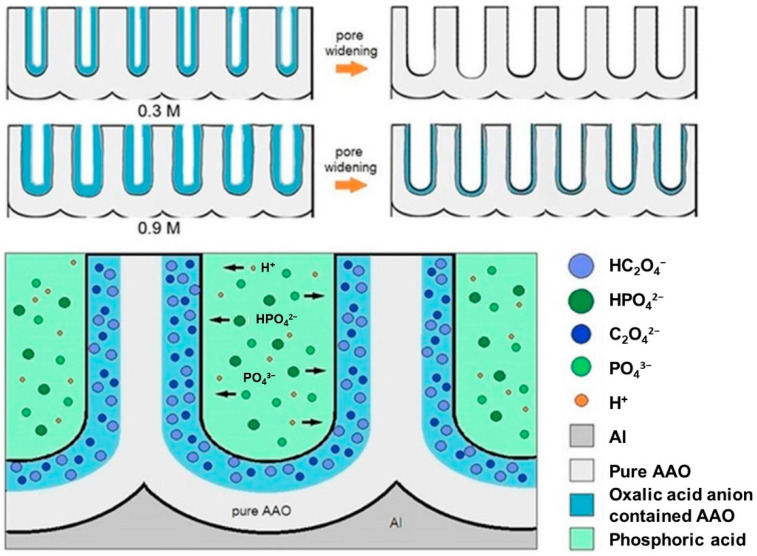
Schematic diagram of AAO pore-widening mechanism in low (0.3 M) and high (0.9 M) oxalic acid concentrations. Reprinted with permission from Ref. [87]. Copyright 2017, Journal of The Electrochemical Society.

In addition to the impact of increased pore size, AAO fabricated in a high-concentration oxalic acid solution exhibits a higher concentration of anions within its pore walls. These anions subsequently influence the rate of wet etching or pore widening. Figure 18 illustrates a conceptual diagram of mechanisms for acid anion contamination in AAO formed in representative low (0.3 M) and high (0.9 M) concentrations. Both situations show that AAO pore walls contain a certain degree of acidic anion contamination. During the anodization process, oxalic acid dissociates into cations (H^+^) and anions (C_2_O_4_^2−^ or HC_2_O_4_^−^). The concentration of dissociated ions is directly proportional to the oxalic acid concentration, indicating that higher oxalic acid concentrations result in a greater number of ions transported, establishing a steeper concentration gradient or driving force, leading to a larger influx of anions diffusing into the AAO pore walls. Based on the inverse behavior of pore evolution in AAO before and after pore widening from low to high oxalic acid concentrations, oxalic acid anions within AAO influence the etching rate of AAO. The etching rate of AAO is significantly reduced at 0.9 M due to the presence of C_2_O_4_^2−^ anions embedded in the AAO walls. As shown in Figure 18, weak phosphoric acid (H_3_PO_4_) in the aqueous solution primarily dissociates into H^+^, H_2_PO_4_^−^, HPO_4_^2−^, and PO_4_^3−^ ions, which must diffuse and migrate to the AAO surface pore walls to undergo etching reactions. During the diffusion of phosphoric acid ions, the negatively charged oxalic acid anion contamination layer interacts with phosphoric acid ions through attraction (H^+^) or repulsion (H_2_PO_4_^−^, HPO_4_^2−^, and PO_4_^3−^), reducing the rate of phosphoric acid ion migration, solubility, and etching rate. The higher the concentration of oxalic acid anions, the lower the etching rate of AAO pore walls in phosphoric acid.

### 3.3. One-Step Anodization Method at 25 °C

**Figure 19:**
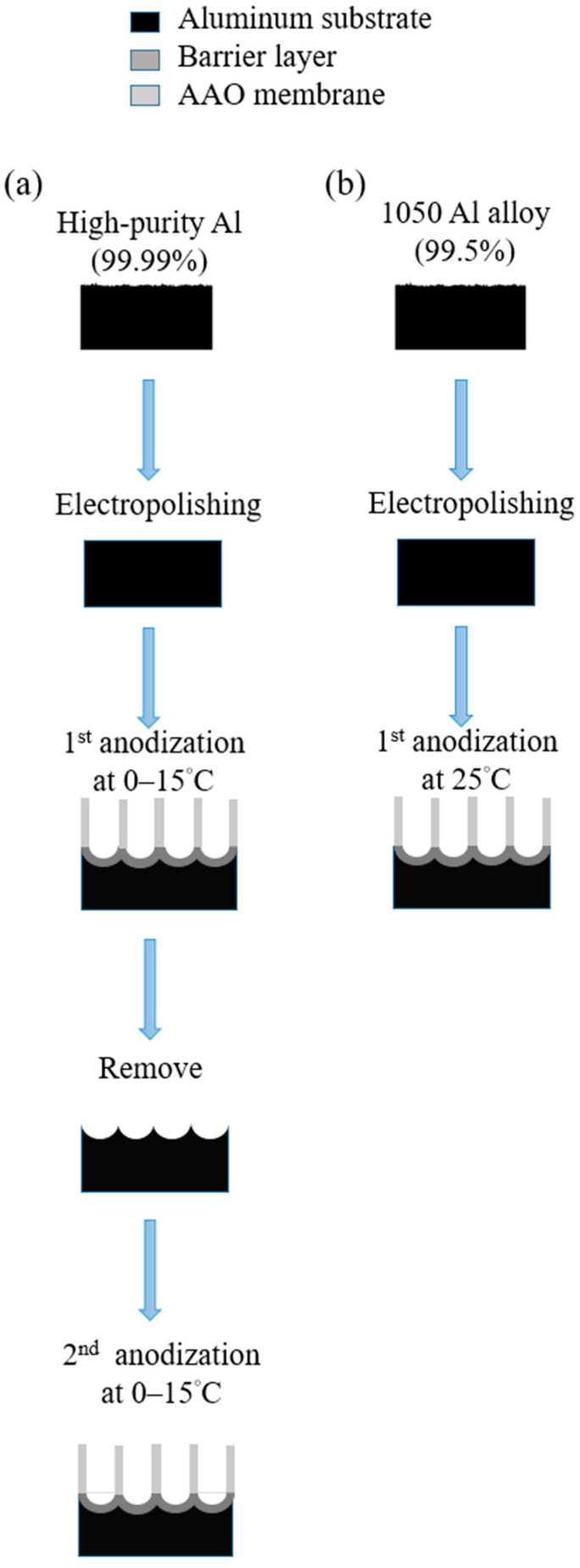
The process flow of (**a**) traditional 2-step process under low temperature of 0–15 °C and (**b**) one-step process at 25 °C.

Figure 19 provides a comparison between one-step and two-step AAO processes. The one-step AAO process eliminates the removal and second-step growth, thereby serving as a promising approach for cost and time reduction. Currently, the industrial dyeing process has adopted the one-step AAO procedure, significantly enhancing the efficiency of fabrication. Further, researches for some applications such as humidity sensors [45] and SERS [120], focusing more on overall-performance-oriented applications, are suitable for the one-step process. Conversely, applications requiring precise structural control still find the two-step process more comprehensive, such as nanomaterial synthesis or nanopattern transfer.

**Figure 20:**
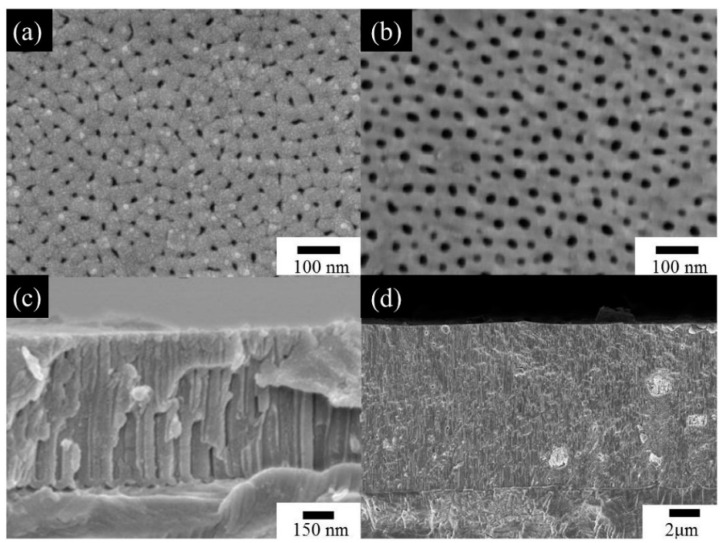
The SEM images of top views (**a**,**b**) and cross-section views (**c**,**d**) for comparison of AAO fabricated at 5 °C (**a**,**c**) and 25 °C (**b**,**d**). Reprinted with permission from Ref. [141]. Copyright 2017, Elsevier.

Figure 20 illustrates a comparison of SEM micrographs of AAOs produced from AA5052 using the HPA process for 2 h at 5 °C (Figure 20a,c) and 25 °C (Figure 20b,d) [141]. When AAO was prepared at 5 °C, the average pore diameter was 15 nm and the thickness was 0.9 um. However, when the temperature was raised to 25 °C, the average pore diameter increased to 32.2 nm and the thickness significantly increased to 19.52 um. This demonstrates that as the temperature rises, AAO achieves efficient thickness growth due to faster dissolution and deposition rates. Additionally, the pore diameter and porosity also increase due to the accelerated dissolution rate.

## 4. Sensor Applications

AAO-based sensor applications can be divided into two categories: electrical sensors and optical sensors. The electrical AAO-based sensor is primarily based on two types of capacitance and resistance; usually, a thin metal film is deposited on the surface of AAO as an electrode for humidity [34,44] and gas [89] measurement. Many more publications on pure AAO humidity sensors than gas ones are probably attributed to the high response in sensing humidity. Moreover, the AAO optical sensor with four kinds of mechanisms, i.e., reflectometric interference spectroscopy (RIfS), photoluminescence (PL), surface plasmon resonance (SPR), and SERS, are reviewed with a focus on SERS applications. For SERS application, AAO can be a solid support for coating popular metal nanoparticles or a temporary template for transferring the nanopores into the nanorods or nanowires for detecting substances. More details are described in the following sections.

### 4.1. Electrical Sensors

With the development of micro–nano technology and automated industry, sensor applications have received attention. Humidity sensors play a vital role in industrial development and daily life, encompassing fields such as weather forecasting, agriculture, process control, and materials or food preservation. AAO is one of the most famous ceramic nanomaterial templates with physical and chemical stability. After depositing a thin film of metal on AAO, the capacitor or resistor structure is formed and easy to apply for humidity measurement. This approach has garnered significant research attention in exploring sensor characteristics. Furthermore, the application of AAO humidity sensors involves measurements within a specific area (mm^2^), where the correlation with individual pore characteristics is not prominent. This characteristic allows the process of humidity sensors to be suitable for the one-step AAO fabrication process, which can replace the traditional two-step, low-temperature method.

In terms of the indicators of humidity sensors, two important characteristics are highlighted. One is the sensor’s response or sensitivity, which signifies its capability to differentiate between different humidity levels. In the context of AAO humidity sensors, this is often measured through capacitance, which can be defined as follows:

(8) $$R=\frac{\Delta C}{C_{0}}$$

where ΔC is the capacitance change and C_0_ is the initial capacitance value of low humidity. Secondly, the response and recovery time indicates the measurement speed and the sensor’s ability to adapt to rapid environmental changes.

The sensing mechanism of AAO humidity sensors can be categorized into three main factors: external conditions, nanostructures [38,39,45], and anionic effect [35,36,37,38]. External conditions primarily involve changes in humidity and the application of an external magnetic field [35,36]. The capacitance calculation and the well-known adsorption theory were introduced by Nahar et al. [33,34]. When the humidity is below 45%, water vapor only forms a chemisorbed layer on the surface, resulting in a lower sensor response. On the other hand, when humidity is above 45%, a physically adsorbed layer is formed and the layering of these physical adsorbed layers causes the capacitance value to rapidly increase [34], exhibiting a natural function curve.

**Figure 21:**
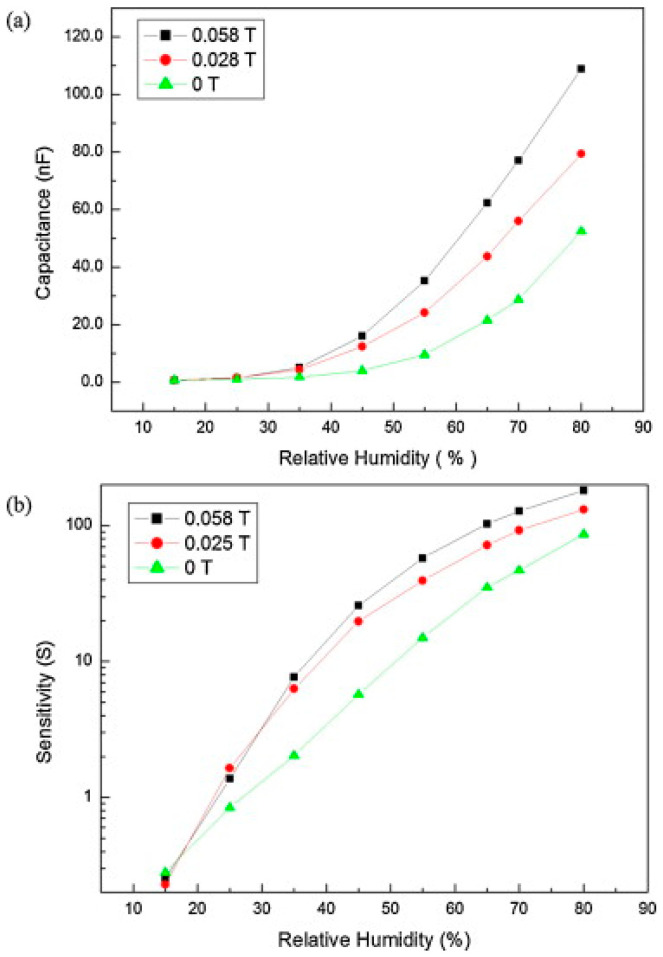
The AAO (**a**) capacitance and (**b**) response under different magnetic field strengths of 0~0.058 T. Reprinted with permission from Ref. [35]. Copyright 2014, Elsevier.

**Figure 22:**
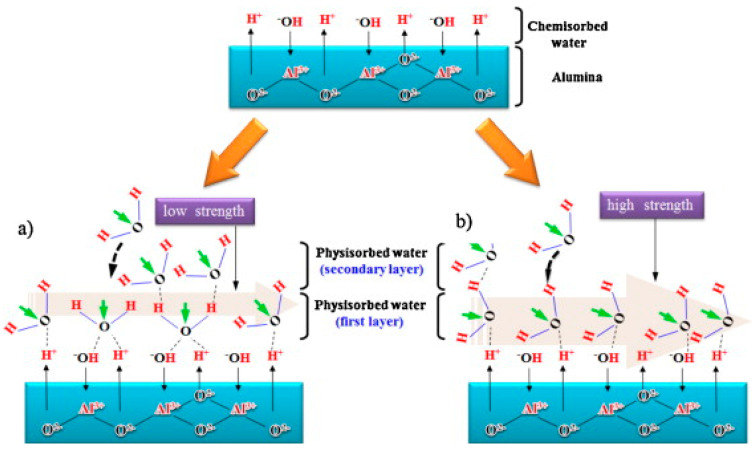
Orientations of water molecule dipoles to the adsorption. (**a**) Low magnetic field and (**b**) high magnetic field. Reprinted with permission from Ref. [35]. Copyright 2014, Elsevier.

The magnetic effect in AAO humidity sensors is illustrated through capacitance measurements in Figure 21 [35]. Figure 21a presents the variation in capacitance with respect to RH values in the range of 15% to 80% under various magnetic field strengths (0~0.058 T). The two-step anodization process was conducted in 0.3 M oxalic acid using HPA at 25 °C for 2 h with a voltage of 40 V. The average sensor response is shown in Figure 21b. In the absence of a magnetic field, the sensor exhibits a capacitance of approximately 0.65 nF at the lowest RH value of 15%. As the relative humidity increases to 45%, the capacitance slightly rises to 4.03 nF, corresponding to a response (R) value of 5.2. Beyond 45% relative humidity, the capacitance experiences a significant increase. At an RH of 80%, the capacitance reaches 52.47 nF, with an R value of 79.7. When the magnetic field increases to 0.058 T, the capacitance notably increases to 16.16 nF, with an R value of 23. As the relative humidity rises to 80%, the capacitance further increases to 108.9 nF, corresponding to a response of 166.5. This novel capacitance behavior indicates that applying a magnetic field can significantly enhance the sensor’s response from low to high humidity levels. These differences arise from the dipole moment of water molecules, leading to a specific arrangement of water molecules, which, in turn, increases the quantity of adsorbed water molecules, contributing to the capacitance value, as shown in Figure 22.

**Figure 23:**
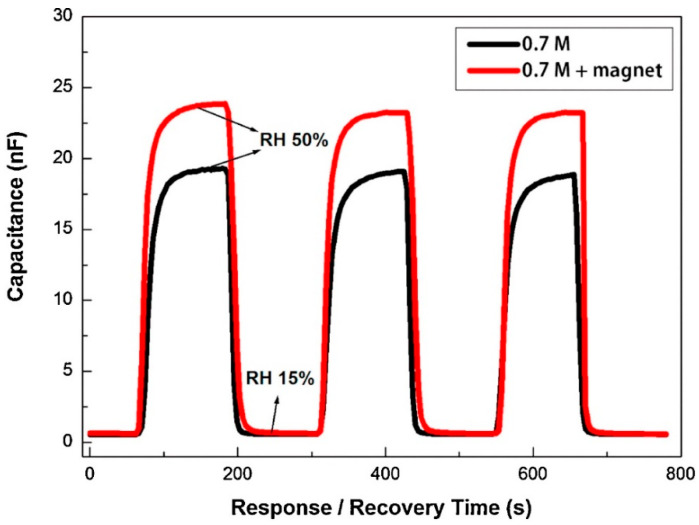
The response–recovery time of the AAO sensors formed at 0.7 M oxalic acid with and without a magnetic field. Reprinted with permission from Ref. [36]. Copyright 2015, Elsevier.

The magnetic field also influences the response–recovery time of the AAO humidity sensor. Figure 23 illustrates the response time (T_rs(10–90)_) and recovery time (T_rc(10–90)_) of the AAO sensor under 0.7 M oxalic acid with and without a magnetic field. In the absence of a magnetic field, the response time is 35 s, while it is 32 s with a magnetic field, which indicates a faster response in the presence of a magnetic field. As these magnetized water molecules tend to align in the specific direction of the field, the presence of a magnetic field makes it easier and faster for water molecule adsorption onto the porous AAO sensor.

In terms of geometrical effect, Kashi et al. [38] studied the sensitivity of AAO by anodizing in phosphoric acid at high voltages of 165–185 V and enlarging the pore size to 107–127 nm. This study aimed to decrease the pore density of AAO while achieving better sensitivity. The improved AAO sensitivity was found to be related to the pore size (Dp) and anodization voltage. Additionally, the number of pores also plays a crucial role. Chung et al. [39] demonstrated that AAO produced from two-step anodization of high-purity aluminum at lower voltages possesses a larger total adsorption area, which can enhance the performance of AAO humidity sensors. A greater total adsorption area can absorb more water, thereby enhancing sensor response. Furthermore, humidity sensors based on AAO typically utilize the traditional two-step anodization process on small areas (<3 cm^2^) of high-purity aluminum (>99.99%) substrates at low temperatures (0–15 °C). However, this often leads to a lower response [40,41,42] and longer response–recovery time [43,44,99]. Non-AAO humidity sensors employing nanofibers, polyaniline (PANI), polyvinyl alcohol (PVA) [142], or nickel sulfide [143] have also been reported, but they also suffer from low strength and extended response–recovery times.

**Figure 24:**
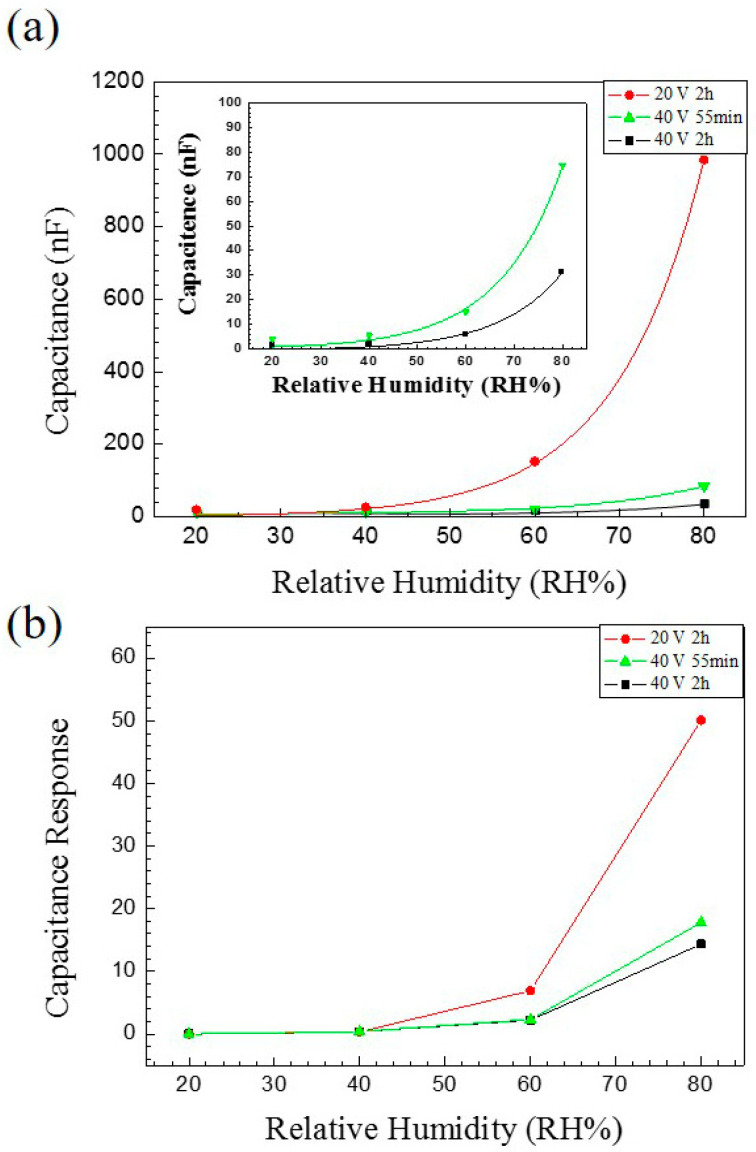
The (**a**) capacitance and (**b**) response vs. relative humidity plot with different voltages and thicknesses of AAO. The two AAO humidity sensors are formed at 40 V for 55 min and 2 h and at 20 V for 2 h; (**b**) the capacitance response of three AAO sensors. Reprinted with permission from Ref. [45]. Copyright 2021, Elsevier.

Figure 24 presents the measurement results of different potentials and thicknesses of AAO. It is evident that the capacitance values of thinner samples are significantly higher, and AAO with lower potentials exhibits a greater response. This phenomenon stems from the contribution of the specific surface area, leading to a response exceeding 5000%.

**Figure 25:**
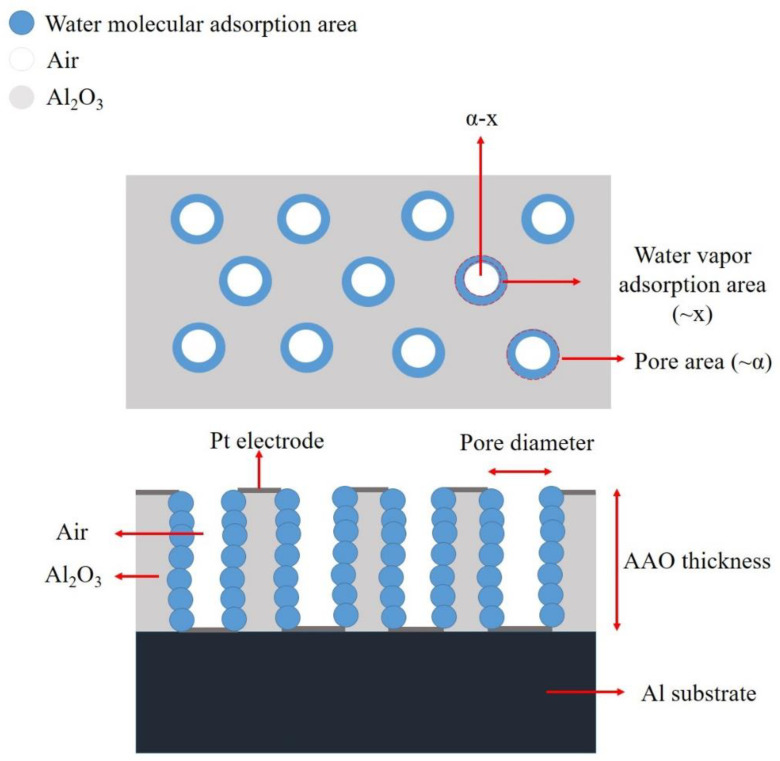
The AAO sensor conceptual diagram: the water molecule adsorption ratio is represented as x, the porosity is represented as α, and the air area ratio is represented as α–x. Reprinted with permission from Ref. [45]. Copyright 2021, Elsevier.

To understand the relationship between AAO nanostructures and the humidity response, we can derive the capacitance formula (Equation (9)) and make assumptions about the water molecules’ adsorption ratio (x) and the porosity (α), as shown in Figure 25. We start with the initial capacitance value (C_0_), which can be considered at RH% = 0% and is assumed to be parallel for capacitors of AAO and air (Equation (10)). The final capacitance at high humidity can then be assumed as the parallel capacitor between water vapor, air, and AAO (Equation (11)).

(9) $$C=\frac{\varepsilon A}{d}$$

(10) $$C_{0}=C_{AAO}+C_{air}=\frac{[\left(\varepsilon_{air}A\alpha +\varepsilon_{{{Al}_{2}O}_{3}}A\left(1-\alpha \right)\right]}{d}$$

(11) $$C_{1}=C_{AAO}+C_{air}+C_{H_{2}O}=\frac{\left[\varepsilon_{air}A(\alpha -x)+\varepsilon_{{{Al}_{2}O}_{3}}A(1-\alpha )+\varepsilon_{H_{2}O}Ax\right]}{d}$$

Therefore, the sensor response (R) can be expressed as Equation (12):

(12) $$\begin{array}{c} R=\frac{\Delta C}{C_{0}}\\ =\frac{\{[\varepsilon_{air}\;A\;(\alpha -x)+\varepsilon_{{{Al}_{2}O}_{3}}A\;(1-\alpha )+\varepsilon_{H_{2}O}\;A\;x]-\left[\left(\varepsilon_{air}A\alpha +\varepsilon_{{{Al}_{2}O}_{3}}A\left(1-\alpha \right)\right]\right\}}{\;[\left(\varepsilon_{air}A\alpha +\varepsilon_{{{Al}_{2}O}_{3}}A\left(1-\alpha \right)\right]}\\ =\frac{\left(\varepsilon_{H_{2}O}\;x-\varepsilon_{air}\;x\right)}{\left[\varepsilon_{air}\;\alpha +\varepsilon_{{{Al}_{2}O}_{3}}\;(1-\alpha )\right]} \end{array}$$

From the formula, it is evident that the final AAO response is not dependent on thickness or area, but rather influenced by the water vapor adsorption ratio and porosity. A higher water vapor adsorption ratio and lower porosity can amplify the response. Consequently, the high surface area structures produced at low voltages can significantly enhance the sensor’s performance.

**Figure 26:**
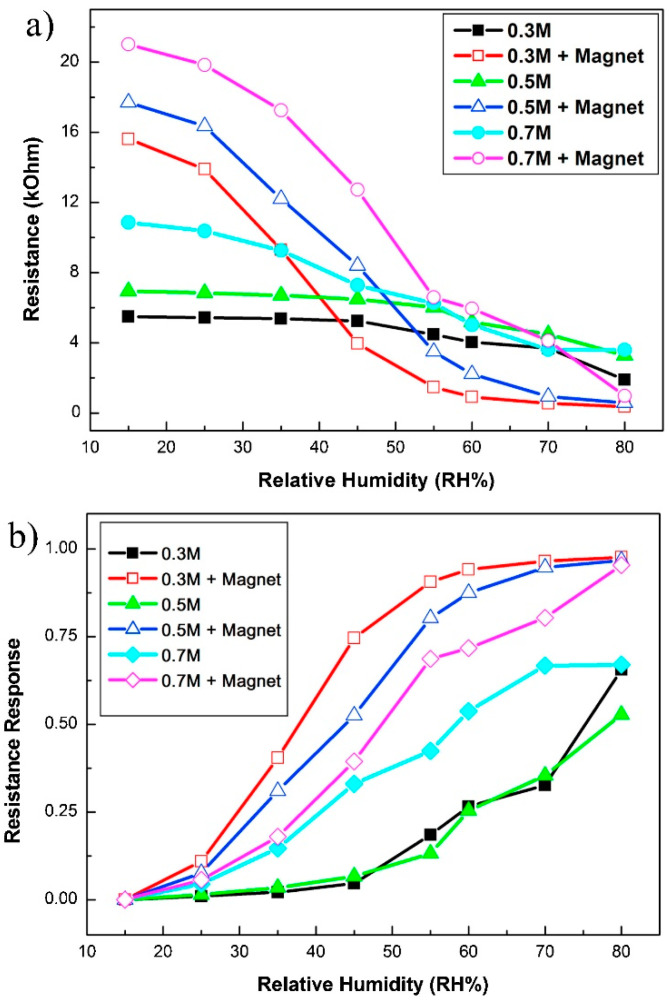
The (**a**) resistance and (**b**) response vs. RH% of the AAO humidity sensor with various oxalic acid concentrations and with or without magnetic field. Reprinted with permission from Ref. [36]. Copyright 2015, Elsevier.

In 2010, He et al. [38] investigated the influence of factors such as anion concentration and annealing on AAO humidity sensors, reporting that anions promote water vapor adsorption, leading to an increase in capacitance values. Figure 26 illustrates the measurement results of AAO humidity sensors prepared in 0.3 M–0.7 M oxalic acid, as proposed by Chung et al. [35,36]. It is evident that higher concentrations, corresponding to greater anion content in AAO, result in more pronounced signal changes. When a magnetic field is applied, a more pronounced response is observed. Additionally, it is worth noting that the resistive measurement method exhibits a better response at low to medium humidity levels, which contrasts with the behavior of capacitive measurements. Consequently, for precise measurements at low humidity levels, resistive AAO sensors could be a promising direction to consider. The AAO electrical sensor of capacitive and resistive type can be further applied to gas sensors [89,144] of organic substances such as ethanol or acetone. However, the greater number of publications on pure AAO humidity sensors than gas ones is probably due to the high response of sensing humidity. This will be an important research direction for AAO sensors in the future.

Table 1 lists a performance comparison between AAO-based humidity sensors [34,35,37,40,41,42,43,44,45] and some non-AAO sensors [142,143]. The traditional AAO humidity sensors are synthesized by two-step anodization from high-purity aluminum at low temperature or by the sputtering method. However, AAO humidity sensors have the potential to shorten the process through one-step anodization. Furthermore, the performance of traditional AAO sensors is better in terms of lower response and longer response–recovery time, but improvements can be achieved through external conditions, nanostructures, and the anionic effect mentioned above.

### 4.2. Optical Sensors

Optical sensors are devices that transform the interaction between substrates and analytes into an optical signal, which is a technique that has been widely applied in chemical trace detection with label-free, fast, and non-invasive advantages. Common signal conversion technologies in optical sensors include reflection interference spectroscopy (RFIS) [102,103,104,105], photoluminescence spectroscopy (PL) [106,107,108,109], surface plasmon resonance (SPR) [110,111,112,113,114], and SERS [115,116,117,118,119,120,121]. AAO is an outstanding substrate for various optical sensing devices with the advantages of nanoscale pores, self-assembly properties, controllable geometry, and good biocompatibility. The optical sensors based on AAO substrates are described in the following sections and summarized in Table 2 with four different mechanisms: RIFS, PL, SPR, and SERS.

RIfS is a useful optical method for sensing the substances, especially biomolecules, which is caused by the interference of light reflected from the top and bottom of the film [106]. The interference spectroscopy depends on the effective optical thickness (EOT), which can be calculated by Equation (13):

EOT = 2 n_eff_ d cosθ = mλ (13)

where EOT is the effective optical thickness, n_eff_ is the effective refractive index of AAO, d is the thickness of the AAO, m is the order of the interference, and λ is the wavelength of the light. After adding the analyte on the AAO film, a change in the EOT causes a shift of the interference spectroscopy. In general, to increase the sensitivity and selectivity of the measurement, the binding reaction between the analyte and the film is essential [106]. The nanostructured porous films compared with the planar polymer films have higher surface area for increasing the sensitivity [107].

Recently, Feng et al. presented an AAO-based RIFS biosensor to detect the different plant hormones including ABA, SA, auxins, cytokinins, and gibberellins by coating an aptamer on the AAO nanofilm [102]. Nemati et al. presented bilayered AAO films with hierarchical funnel-like structures for optimizing the optical sensing performance toward multi-analyte biosensing [103]. Kaur et al. combined self-assembled glutaraldehyde-cross-linked, double-layered, polyethylenimine (PEI-GA-PEI)-modified AAO interferometers and reflectometric interference spectroscopy (RIfS) as an optical sensing system for detecting ionic copper in environmental waters [104]. Kapruwan et al. exploited the optical properties of AAO gradient index filters, which have been used to measure the release dynamics of the cargo molecule in real time [105]. PL refers to light emission after the absorption of photons, which causes photon excitation and produces the electronic transition. The absorbed light causes the electrons of the material to jump to a higher state and then return back to a lower energy level with a light emission [107]. Recently, Pla et al. presented a biosensor for the fluorogenic identification of Staphylococcus aureus cells using two-step vertical AAO, which were filled with rhodamine B and capped with the aptamers. After the Staphylococcus aureus cells interact with the aptamer, the fluorogenic dye is released [108]. Malinovskis et al. produced aligned ZnO nanorods with the property of PL, which was embedded in two-step vertical AAO. The analyte, human vascular endothelial growth factor, decreased the intensity of PL by the ZnO nanorods and can be detected [109].

Surface plasmon resonance (SPR) is the oscillation of electrons on a metallic surface stimulated by an extra incident light. The analyte is detected based on the changes in the absorbance of SPR of the substrate [110]. Furthermore, AAO can be used to fabricate some nanostructure to produce localized surface plasmon resonance (LSPR). Compared with SPR, LSPR is more easily excited, which means complex optical configurations are unnecessary. Thus, some LSPR sensing researches using simple, even portable, optical devices have been reported [110,111,112,113,114]. Lednický et al. detected label-free DNA of Giardia lamblia by measuring the red-shift in the LSPR absorbance spectrum. The LSPR is caused by the Au nanoparticles synthesized on the nanobowled barrier of the aluminum substrate after removing the AAO layer [110]. Lv et al. fabricated ordered nanoellipsoid arrays to generate LSPR for sensing the protein CD63. The ordered nanoellipsoid arrays were fabricated with the two-step, through-pore AAO film as a shadow-mask for evaporating Au [111].

Recently, SERS has been the most-published topic and can be applied for trace detection of various substances. Therefore, this section is focused on SERS sensing applications based on AAO-related substrates. SERS is a sensitive technique for detecting trace substances in industry, environment, wastewater, and air pollution. The enhancement comes from the overall effect of the electromagnetic mechanism (EM) and the chemical mechanism (CM). The EM is attributed to the local electrical field enhanced by the plasma resonance, and the CM is attributed to the electrons transfer between the analyte and the SERS substrate [120]. Here, the SERS substrates majorly based on the EM are discussed because the enhancement of EM is substantially higher than that of CM. The porous materials with properties of simple manufacturing and high aspect ratio are regarded as good candidates for forming the nanostructures of SERS substrates. Therefore, AAO with tunable nanopores is a popular material for fabricating the SERS substrate with various approaches. The current reported AAO-based SERS substrates can be divided into MNPs in the AAO pores [115,116,117], using AAO as a mold for fabricating the metal nanostructure array, and the metal film on the nanopores. First, AAO can be the support for the nanoparticles: Lin et al. used a drop-dry approach to deposit gold nanobipyramids (Au NBPs) into vertical two-step AAO pores as the SERS substrate to detect Aflatoxin B1. For Aflatoxin B1 sensing, the Au NBPs–AAO substrate has a corresponding linear range from 1.5 μg/L to 1.5 mg/L and an LOD of 0.5 μg/L [115]. Chen et al. decorated the vertical two-step AAO with Au-Ag core–shell particles as SERS nanotags for sensing the inflammatory biomarkers such as C-reactive protein, interleukin-6, serum amyloid A, and procalcitonin. The analyte was captured in the nanopores of the AAO film, which shows different SERS performance with the pore diameter of 140 to 390 nm. The SERS intensity increased as the pore diameter of AAO increased from 140 to 350 nm, and the intensity of the spectra decreased as the pore diameter increased to 390 nm. The increased intensity of SERS with increased pore diameter can be attributed to the red-shift of the absorption peak and leads to high excitation efficiency because of the overlap between the absorption peak and the incident light. On the other hand, the decreased intensity of SERS can be attributed to the decreased coupling effect between nanopores with too-large a pore diameter. The larger pore diameter leads to a shorter time for the sample to enter the reaction for antigen–antibody binding in the nanochannels [118]. Tezcan et al. demonstrated flower-shaped Au nano clusters on the AAO by using pitting corrosion to release the Al ions and, thus, reduce the gold ions. In contrast, Au particles deposited as a bulk on the bare aluminum foil. The nanoporous structure leads to the aggregated Au nano clusters being accompanied with many hot spots. An LOD of 1.03 ppm was performed by using the flower-shaped Au nano clusters as the SERS substrate to detect nitrate in the water [117]. Wang et al. reported the one-dimensional defective photonic crystals (PCs) based on bamboo-like AAO fabricated by periodic pulse anodization. As mentioned in Section 1, the pore diameter and interpore distance of AAO increase with the applied voltage during the anodization. The bamboo-like AAO can be fabricated as PCs by periodically altering between high and low voltages. After loading the Ag MNPs to produce the SPR, the SERS performance of the AAO PCs was tested by rhodamine B (rhB), and the LOD of the rhB based on the AAO PCs reached the order of 10^−10^ mol/L [90].

**Figure 27:**
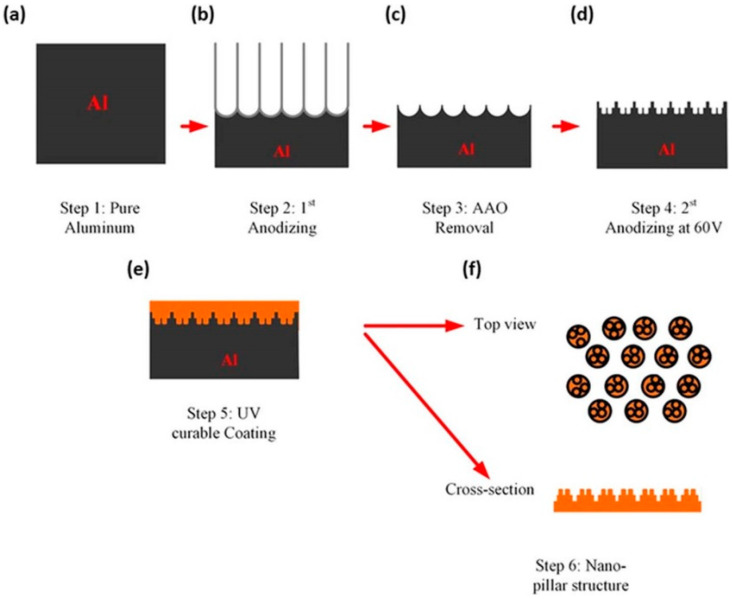
The schematic of fabricating the nanorods array using the 2-step branched AAO, which involves (**a**) the pure aluminum, (**b**) applying the first anodization on the aluminum to form the 1-step AAO film, (**c**) removing the 1-step AAO film, (**d**) applying the second anodization on the aluminum to form the branch AAO, (**e**) coating the UV-curable resin on the AAO, and (**f**) the top view and cross-section of the nanopillar of the UV-curable resin.

**Figure 28:**
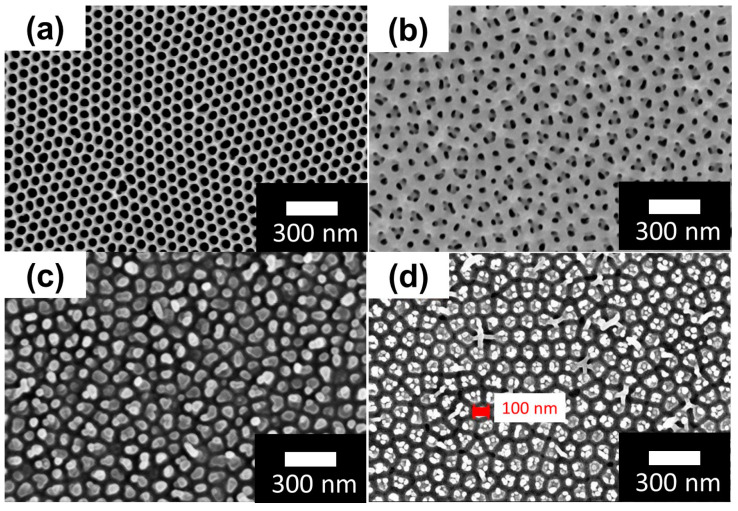
Top-view SEM micrographs of AAO with (**a**) vertical and (**b**) branched pores, and the nanorods array fabricated by (**c**) vertical and (**d**) branched AAO.

Second, AAO can be the mold or the mask to fabricate the nanoparticles, nanowires, and nanorods. Chung et al. demonstrated the UV-curable resin nanorods array deposited with Ag MNPs as the SERS substrate. The UV-curable resin was filled into the nanopores of the two-step vertical and branched AAO to transfer the morphology of the nanopores to the UV-curable resin and generate the nanorods array. The process of fabricating the nanorods array with AAO template is shown in Figure 27 [118]. The SEM micrographs of the vertical and branched AAO nanorods are shown in Figure 28. The nanorods array fabricated by the branched AAO provides smaller and more numerous hot spots. Sanguansap et al. fabricated striped and porous striped gold–silver alloy nanowires (Au-Ag alloy NWs) with commercial, through-pore, vertical AAO. The thickness of the NWs can be simply controlled by adjusting the deposition pulse time. Using the Au-Ag alloy NWs as the SERS substrate, the detection of β-hydroxybutyric acid (BHB) was performed with a low detection limit of about 11 nM [119].

**Figure 29:**
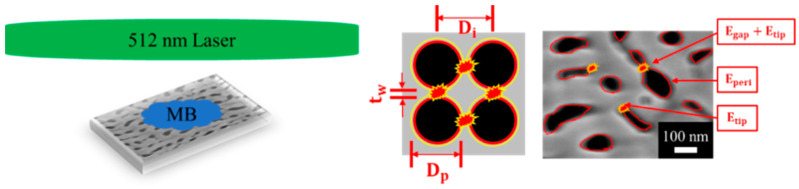
The mechanism of using irregular AAO nanopores as the SERS substrate. Reprinted with permission from Ref. [120]. Copyright 2021, Elsevier.

**Figure 30:**
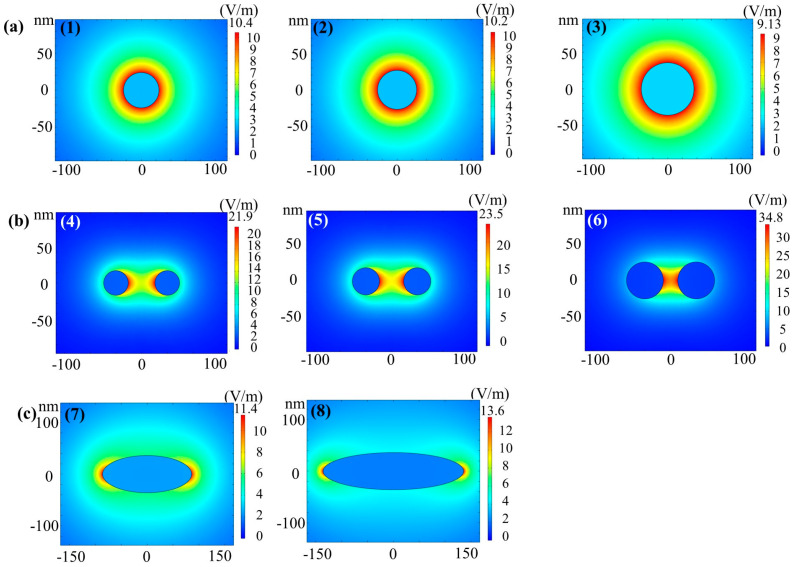
Simulating the electric field using COMSOL^®^ for (**a**) single pores with a diameter of (1) 46, (2) 53, and (3) 72 nm; (**b**) the gap of (4) 54, (5) 47, and (6) 28 nm between two pores; and (**c**) the ellipses with the semi-major axis and semi-minor axis of (7) 86, 36 and (8) 136, 36, respectively. Reprinted with permission from Ref. [120]. Copyright 2021, Elsevier.

Recently, Chung et al. presented a pore peripheral plasmonic mechanism using one-step irregular AAO composited with Pt metal film. As Figure 29 shows, the plasmonic oscillation around the edge of the pore is denoted as E_peri_; the extra hot spots from the tiny gap between two neighboring pores or the high-curvature tips are denoted as E_gaps_ and E_tips_, respectively. SERS performance increased with pore-widening time, which provides more E_peri_ and stronger E_gaps_ and E_tips_. This result was simulated using COMSOL^®^, as shown in Figure 30. The LOD of 1 × 10^−9^ M was performed using irregular AAO as the SERS substrate for detecting MB molecules [120]. To fabricate the SERS substrate with high EF, low LOD, good linearity, and good uniformity is a hot topic in this field, and AAO is a good candidate because of the advantages of nanoscale pores, self-organization, controllable pore size, and good biocompatibility and thermal stability. In this section, the recently reported optical sensors based on AAO with various mechanisms and profiles for detecting the different substances are reviewed. Because of the advantages of being label-free, fast, and non-invasive, the optical sensors based on AAO have great potential for various applications in industry, environment, wastewater, and biosecurity [145].

## 5. Conclusions

In this review, we introduce various AAO fabrication processes and nanostructure profiles. The recent advances in AAO electrical and optical sensors are linked to our environment, product manufacturing, daily life, and safety. Conventional AAO templates can be divided into three categories: the two-step process, pretextured method, and sputtering Al on other materials to integrate AAO with different substrates. Until now, growing AAO directly from aluminum substrates using the two-step DCA process at a low temperature (−5~15 °C) has been the mainstream method. However, to improve the efficiency of AAO preparation, HA was proposed and has gradually improved the AAO growth rate. Furthermore, an effective method of HPA with normal-positive and small-negative voltages is effective for AAO synthesis at relatively high temperatures of 20–30 °C and voltages of 40–60 V for enhancing the performance of the AAO structure for both cheap low-purity (99%) and costly high-purity (99.997%) aluminum materials. The performance of AAO applications are related to the sensing substance and the pore geometry that is concerned with the materials composition, electrolyte and anodizing parameters. The effect of different fabrication parameters on the AAO pore size and the profiles for various application fields is investigated. In brief, there are currently highly efficient preparation methods available for the structural control of AAO. However, to adjust different structures to meet various applications is a future direction and challenge. AAO sensor applications can be divided into two categories: electrical sensors and optical sensors. The electrical AAO sensor that is primarily based on both types of capacitance and resistance usually deposits a thin metal film on the surface of AAO as an electrode for humidity and gas measurement. The geometry, anion, and magnetic mechanism are reviewed in detail for fabricating a high response– and a short response–recovery time AAO humidity sensor. In the field of electrical sensing, moving towards organic gas sensing to contribute to environmental safety is a future direction. Although there are a few papers for the organic gas investigation, the selectivity of different organic targets remains a major challenge. In addition, the AAO optical sensor with four kinds of mechanisms, i.e., interference, photoluminescence, surface plasma resonance, and SERS are reviewed, with a focus on SERS applications. To fabricate the SERS substrate, AAO can be a solid support for coating the popular metal nanoparticles or a temporary template for transferring the nanopores into the nanodots or nanowires for detecting substances. AAO–SERS technology has the potential to expand into food safety examination, covering areas such as agriculture, water quality, and additives for food. However, achieving accurate determination in situations with significant interference and establishing pretreatment methods remain unresolved issues. Although there are still challenges for AAO sensors, the widely tunable pore size of AAO and its modification for high-performance sensors will play a crucial role for the sensors used in Internet of Things in our living environment and promote our quality of life.

## Figures and Tables

**Table 1:** Comparison of humidity sensor fabrication and performance.

Reference	Fabrication Method/Time	Signal Intensity (RH%)	Response (ΔC/C_0_)	Response–Recovery Time
[34]	AAO/NA	0.08–20 nF (RH5%–95%)	About 20,000%	NA
[35]	AAO prepared by oxalic acid from high-purity Al/4 h	0.65–108.9 nF (RH15%–80%)	16,650%	NA
[37]	AAO prepared by oxalic acid from high-purity Al at −5 °C/NA	1.5–60 nF (RH5%–95%)	About 3900%	NA
[40]	AAO prepared by oxalic acid and pore widening from high-purity Al/6 h	1.8–16 nF (RH5%–75%)	793.02%	NA
[41]	Al sputtering on paper and anodized by phosphoric acid from high-purity Al/NA	NA (RH20%–80%)	About 500%	NA
[42]	Spin coating polymer material on AAO/NA	10–38 pF (RH20%–90%)	280%	NA
[43]	Sputtered Al for AAO on Si from high-purity Al/NA	2.08–2.17 pF (RH30%–90%)	About 4.4%	289 s/286 s
[44]	AAO prepared by oxalic acid and pore widening from high-purity Al at 15 °C/8 h	NA	About 4830%	18–188 s/NA
[99]	Silica and poly(3,4-ethylenedioxythiophene) composites/NA	About 20–1000 pF (RH11%–95%)	About 4000%	77 s/30 s
[142]	carbon nanofiber (CNF) and nanofibrillated cellulose (NFC)/27 h	About 231-3290 pF (RH40%- 100%)	About 100%	41 s/50 s
[143]	bis(4-benzylpiperazine-1-carbodithioato-k2S, S′)nickel(II) complex/NA	15.95 pF–38.1 pF (RH30%–90%)	About 138%	25 s/30 s
[45]	AAO prepared by oxalic acid/2 h	19.25–984.26 nF (RH20%–80%)	5013%	Below 10 s/10 s

**Table 2:** Comparison of recent optical sensors based on AAO with the mechanisms of RIfS, PL, SPR, and SERS.

Ref.	Technique	Analyte	LOD	Substrate
[102]	RIfS	Plant hormones (ABA, SA, auxins, cytokinins, gibberellins)	0.1 μm	One-step vertical AAO on ITO glass chip
[103]		Quercetin	0.14 mg/mL	Two-step bilayered or funnel-like NAA structures
[104]		Cu^2+^	0.007 ppm	Two-step vertical AAO
[105]		Drug release	-	One-step bamboo-like AAO fabricated by Sinusoidal anodization
[108]	PL	Staphylococcus aureus Cocaine	0.5 μm	Two-step vertical AAO
[109]		Vascular endothelial growth factor (VEGF)	1 ng/μL	Two-step vertical AAO filled with ZnO
[110]	SPR/LSPR	Label-free DNA	5 nm	Nanobowled AAO barrier
[111]		Transmembrane protein CD63	1 ng/mL	Gold nano array fabricated using 2-step vertical AAO as a mask
[112]		IgA	10 ng/μL	Gold-capped 2-step vertical AAO
[113]		Label-free DNA	10 nM	Gold nanoantenna array fabricated using 1-step vertical AAO as a mask
[114]		Cell interleukin-6	10 ng/mL	Nanoimprinting cyclo-olefin polymer (COP) using 2-step vertical AAO mold
[115]	SERS	Aflatoxin B1	0.5 μg/L	Bipyramid-like nanoparticles in 2-step vertical AAO
[116]		C-reactive protein, interleukin-6, serum amyloid A, and procalcitonin	53.4, 4.72, 48.3, and 7.53 fg/mL	Gold–Silver core–shell nanoparticles on commercial AAO
[117]		Nitrate ion	1.03 ppm	Gold nanoparticle clusters on 1-step AAO layer
[118]		Methylene blue	-	UV-curable resin nanorod arrays using the 2-step branched AAO as a mold
[90]		Beta-hydroxybutyric acid	11 nM	Striped Au-Ag nanowire fabricated using vertical AAO as a shadow mask
[119]		Rhodamine B	1 × 10^−10^ mol/L	Ag-loaded bamboo-like AAO as 1D photonic crystal and defective photonic crystals
[120]		Methylene blue	1 nM	Irregular 1-step AAO pores
[121]		Melamine	0.05 ppm	Irregular 1-step AAO pores in 1–2 μm cavities

## Data Availability

Data are the coauthors’ research results and schematic drawing.
